# Treatment Patterns, Characteristics, and Probable Acute Medication Overprescription Among Patients With Migraine in Japan: A Retrospective Cross-Sectional and Longitudinal Analysis of Health Insurance Claims Data

**DOI:** 10.7759/cureus.75928

**Published:** 2024-12-18

**Authors:** Masahito Katsuki, Yasuhiko Matsumori, Taisuke Ichihara, Yuya Yamada, Keiichi Kaneko, Yasushi Kobayashi, Shin Kawamura, Kenta Kashiwagi, Akihito Koh, Tetsuya Goto, Kazuma Kaneko, Naomichi Wada, Yoshiki Hanaoka, Fuminori Yamagishi

**Affiliations:** 1 Physical Education and Health Center, Nagaoka University of Technology, Nagaoka, JPN; 2 Department of Neurosurgery, Japanese Red Cross Suwa Hospital, Suwa, JPN; 3 Department of Neurosurgery, Itoigawa General Hospital, Itoigawa, JPN; 4 Neurology, Sendai Headache and Neurology Clinic, Sendai, JPN; 5 JAST Lab, Japan System Techniques Co. Ltd. (JAST), Tokyo, JPN; 6 Neuroscience, Japan Drug Development and Medical Affairs, Eli Lilly Japan K.K., Kobe, JPN; 7 Department of Neurology, Itoigawa General Hospital, Itoigawa, JPN; 8 Department of Neurology, Japanese Red Cross Suwa Hospital, Suwa, JPN; 9 Department of Neurosurgery, Shinshu University School of Medicine, Matsumoto, JPN; 10 Department of Surgery, Itoigawa General Hospital, Itoigawa, JPN

**Keywords:** areal deprivation index (adi), calcitonin gene-related peptide-related monoclonal antibodies (cgrp-mabs), medication-overuse headache (moh), prophylaxis, specialists

## Abstract

Objective

This study aimed to investigate prescription patterns for migraine patients aged 18 years and older using the REZULT database, managed by Japan System Techniques Co., Ltd. in Tokyo, Japan.

Methods

The study used data from employee-based insurance claims within the REZULT database and comprised two components. In the first part, a cross-sectional analysis (Study 1) was conducted to determine the rate of acute medication overprescription among patients diagnosed with migraines in 2020. Overprescription was defined as receiving at least 30 tablets within 90 days for triptans, combination nonsteroidal anti-inflammatory drugs (NSAIDs), or multiple types of medications, or at least 45 tablets for single NSAIDs within the same period. The second component, Study 2, involved a longitudinal analysis, tracking patients for more than two years from their initial migraine diagnosis, covering the period from July 2010 to April 2022. The number of prescribed tablets was recorded every 90 days.

Results

In Study 1, out of 3,300,705 patients evaluated in 2020, 66,428 (2.01%) were diagnosed with migraines. Of these, 41,209 (62.04%) received acute medications. Overprescription was observed in 9,280 patients (22.52%) for single NSAIDs and in 2,118 patients (5.14%) for triptans. Additionally, 6,412 patients (15.56%) received prophylactic treatment. In Study 2, among 6,840,618 patients followed for more than two years, 296,164 (4.33%) had a persistent diagnosis of migraines over the study period. Overprescription rates were 23.20% (68,704 patients) for single NSAIDs and 3.97% (11,755 patients) for triptans, while 48,886 patients (16.51%) received prophylactic medication at least once. The treatment patterns were influenced by socioeconomic factors, such as the area deprivation index, and the distribution of headache specialists.

Conclusions

Our assessment of real-world data revealed that prophylactic medications are underprescribed, while moderate to high rates of acute medication overprescription were noted among migraine patients.

## Introduction

Migraine is a common public health issue, managed through acute medications to address headache attacks and preventive medications aimed at decreasing the frequency and intensity of these attacks [1]. When severe headache disorders are managed inadequately without the use of preventive treatments, there is an increased risk of developing chronic migraines, treatment resistance, additional health complications, and medication-overuse headache (MOH) [2,3]. Overprescription can be related to MOH and result in chronic migraine development [4]. Therefore, it is very important not only to educate people about headaches [5,6] but also to properly diagnose and treat them by consulting a headache specialist. However, there is a shortage of headache specialists, leaving the management of primary headaches largely to general physicians globally [7-10]. This has led to major concerns about the improper and excessive prescription of acute medications, often without sufficient use of prophylactic treatments.

There can be large differences in treatment patterns depending on the region where patients and doctors live as headache specialists are unevenly distributed in Japan. Additionally, the patients’ and doctors’ social networks can also affect the treatment pattern. Thus, community situation, as a more comprehensive external factor including quality of health service and social behavior of a group of residents, may also affect treatment patterns. Area socioeconomic ‘deprivation’ is defined as a state of observable and demonstrable disadvantage relative to the local community or a wider society or nation to which an individual, family, or group belongs. The area deprivation index (ADI) has been widely used as a socioeconomic indicator and is related to disease outcomes [11-13], but there are no reports on the association between ADI and migraine. Furthermore, although many concerns exist about headache prescribing patterns, there is scarce real-world data in Japan examining the extent to which inappropriate prescribing patterns are being practiced.

Recently, research based on National Health Insurance claims data, encompassing large patient datasets as real-world data, has been published [14-19]. However, such research efforts in Japan are limited [20-23]. Given that big data research can reveal issues related to inappropriate prescriptions by physicians in Japan, we analyzed a health insurance database to understand migraine treatment patterns. Specifically, we used real-world data to assess both the overprescription of acute medications and the use of prophylactic treatments, in the context of the ADI. This study stands out from prior research due to its large sample size, cross-sectional analysis of migraine prescription patterns in Japan, and the longitudinal approach, tracking changes in prescription trends and the trend of overprescriptions and prophylaxis use for two years from the initial headache diagnosis.

We have previously published articles investigating “headache” diagnosed patients’ treatment patterns in the Japanese National Health Insurance Claim dataset. This study's procedures and statistical methods are the same as those in our previous reports [24,25]. However, there is one difference in the current study in that the diagnosis name was extracted from patients with “migraine,” not “headache.” “Headache” can include secondary headaches caused by trauma or colds, which may introduce a bias; however, by relying only on “migraine,” this bias can be considerably reduced. This constitutes an essential point of divergence between this study and the previous studies.

## Materials and methods

General information

In Japan, all residents are required to be covered by health insurance, provided either through employer-based or community-based insurers, with the government establishing the fee schedules for medical services [20]. The REZULT database, managed by Japan System Techniques Co., Ltd. in Tokyo (https://www.jastlab.jast.jp/rezult_data/), contains insurance claim data for over eight million patients, including those aged under 18 years. It includes more than 420 million claims from 147 insurance companies, enabling continuous tracking of prescriptions and treatments for up to 12 years. Most patients in the database are of working age. It records claim dates, disease names, patient demographics (age and sex), medical treatments, prescription details, and the medical institutions involved. The medical departments listed are based on the physicians’ main specialties rather than board-certified specialties. Each patient is assigned an anonymous unique identification number, ensuring consistent prescription tracking over time.

In Japan, both single and combination analgesics can be obtained over-the-counter (OTC). However, triptans and preventive medications for migraines cannot be sold OTC and can only be prescribed by doctors to patients with a migraine diagnosis. On the other hand, single and combination nonsteroidal anti-inflammatory drugs (NSAIDs) can be prescribed for headaches even without a migraine diagnosis and are also used for other chronic pain conditions like lower back pain. However, the database used in this study does not specify the conditions for which the medications were prescribed.

According to the Clinical Practice Guideline for Headache Disorders 2021, prophylactic treatments included the prescription of lomerizine, propranolol, valproic acid, amitriptyline, and verapamil [1]. The prophylactic medications are indicated as follows: “(1) for patients who have migraine attacks two times or more per month; (2) for patients who have headaches that interfere with daily life three times or more per month; and (3) when migraine-induced disability in daily living remains with acute treatment alone, when acute treatment drugs cannot be used, and for special types of migraine with a risk of causing permanent neurological defects.”

Calculation of area socioeconomic status and headache specialists’ distribution in Japan

ADI representing socioeconomic status in Japan consists of weighted sums of census-based variables. We retrieved variables from the 2020 Japanese Census and calculated the ADI of each prefecture with the following equation [11,26].

\[ \begin{aligned} ADI = & \; 2.99 \times \text{proportion of old couple households} \\ & + 7.57 \times \text{proportion of old single households} \\ & + 17.4 \times \text{proportion of single-mother households} \\ & + 2.22 \times \text{proportion of rented houses} \\ & + 4.03 \times \text{proportion of sales and service workers} \\ & + 6.05 \times \text{proportion of agricultural workers} \\ & + 5.38 \times \text{proportion of blue-collar workers} \\ & + 18.3 \times \text{unemployment rate} \end{aligned} \]

**Figure 1:**
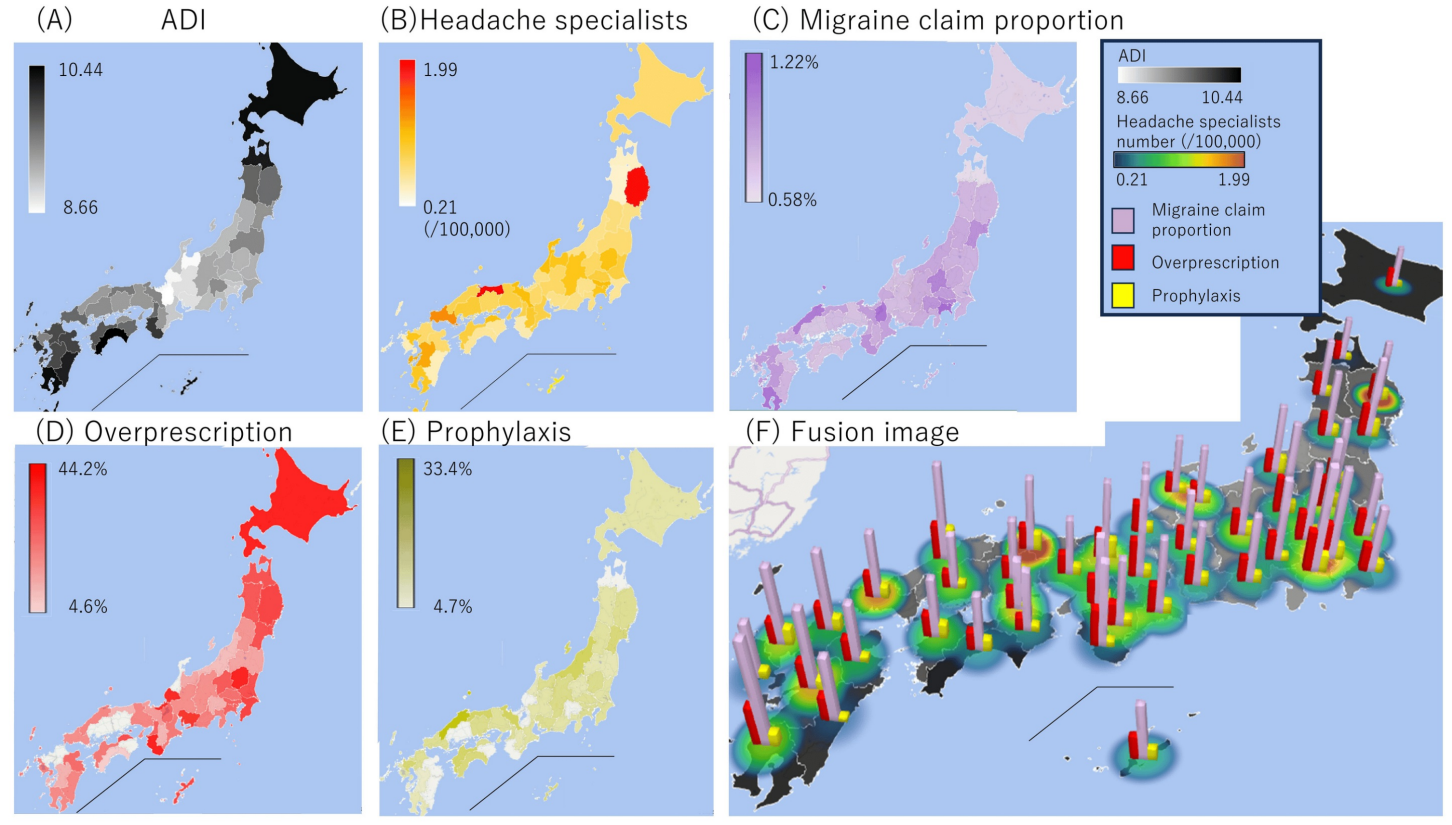
Regional characteristics (A) Area deprivation index (ADI) is shown. Higher ADI and black areas indicate higher poverty. (B) The number of headache specialists per 100,000 people is shown. The redder the color, the greater the number of headache specialists per population. (C) The percentage of patients with a prescription for migraine out of all REZULT database patients is shown by region. The darker the color, the greater the percentage of migraine patients with prescriptions. (D) The percentage of overprescribing among all prescriptions for migraine patients in the region. (E) The percentage of prophylactic medication among all prescriptions for migraine patients in the region. (F) Fusion image of all the five indexes

The ADI of each prefecture is shown in Figure 1A.

The ADI for each prefecture was used as a variable in studies 1 and 2, which are described below. In cases where the patient visited more than one medical facility, the ADI for the region of the medical facility where the prescription or diagnosis was first made was used. The number of headache specialists (specialists/100,000 population) per prefecture was calculated from the list of headache specialists (https://www.jhsnet.net/ichiran.html).

Study 1 (cross-sectional study): prescription pattern for patients with migraine

The data for this study were obtained from the REZULT database. The inclusion criteria involved selecting data for patients over 17 years old who had a continuous migraine diagnosis (ICD-10 code G43) for one year in 2020 and were prescribed acute medications listed in Table 1.

**Table 1:** Summary of acute and prophylactic medications

Treatment type	Drug name
Acute treatment (this study extracted details of patients who were prescribed)	Combination of nonsteroidal anti-inflammatory drugs
	Single nonsteroidal anti-inflammatory drug
	Triptan
Prophylactic treatment (investigation of the prescription pattern after the above extraction)	Calcium-channel blockers (lomerizine)
	Beta-blockers (propranolol)
	Anticonvulsants (valproic acid)
	Antidepressants (amitriptyline)
	Calcium-channel blockers (verapamil)

This enabled an analysis of the number of patients receiving overprescriptions of acute medications as well as prescriptions for prophylactic treatments. There was no differentiation based on the presence or absence of a headache diagnosis (ICD-10 code R51). The diagnosis was made by the doctors, but it is uncertain that they made the diagnosis appropriately based on the International Classification of Headache Disorders, 3rd edition (ICHD-3). The drug price list codes are presented in the Appendices. Exclusion criteria were not applied due to the study’s reliance on national health insurance claims data.

Acute treatments were defined as prescriptions for single NSAIDs, combinations of NSAIDs, or triptans. Prophylactic treatments included medications such as lomerizine, propranolol, valproic acid, amitriptyline, and verapamil, based on Japanese guidelines [1]. The number of acute medication tablets prescribed from January to December 2020 was counted, and the extent of overprescriptions was assessed. In line with prior research on triptan overuse [14], overprescription was determined according to ICHD-3, defining it as the use of triptans or combination NSAIDs on more than 10 days per month over three months (≥30 tablets/90 days), single NSAIDs on more than 15 days per month (≥45 tablets/90 days), or any combination of single, combination, or triptans (≥30 tablets/90 days) without being specific to a single medication.

Since it was not feasible to determine the exact number of days acute medications were used during the study period, the analysis focused on the "number of tablets per 90 days" rather than the "number of days per month" when acute medications were taken. For each patient, the 90-day period with the highest prescription of acute medications was identified, and the prescribing pattern was analyzed. The tables display prescribing patterns over the 90-day period when the drugs were most frequently prescribed. Prophylactic treatment was defined as "prescribing more than one tablet within a 90-day period." Most prophylactic migraine medications have additional indications, including hypertension, epilepsy, and depression.

Study 2 (longitudinal study): overprescription during the two years from the initial diagnosis

We monitored changes in prescription patterns over a two-year period following the initial migraine diagnosis to identify how many patients received excessive acute medications. The inclusion criteria involved analyzing data from 6,840,618 patients over 17 years old in the REZULT database who could be followed up for more than two years from their initial migraine diagnosis, between July 2010 and April 2022. Given this study used national health insurance claims data, exclusion criteria were not applied.

The number of prescribed tablets was recorded every 90 days from the initial diagnosis, and the proportion of patients with overprescription during the study period was evaluated. We examined age, sex, the timing of the onset of overprescription, the quantity of acute medication prescribed in the first 90 days, and the use of prophylactic medication during that same period. Cox regression analysis was used to determine the factors associated with the duration of overprescription of acute medication. No prescriptions for calcitonin gene-related peptide (CGRP)-related drugs were found in the dataset during this period.

Since analyzing overprescription within 90-day intervals (one term) could potentially overestimate the number of patients affected, a sensitivity analysis was conducted by examining patients who continued to experience overprescription over 180 days (two terms).

Statistical analysis

Normal distribution was confirmed using the Shapiro-Wilk test. Variables with a normal distribution are presented as mean and standard deviation (SD), whereas those with a non-normal distribution are presented as median [interquartile range (IQR). A log-minus-log plot was used to check whether the assumption of proportional hazards was satisfied. A priori statistical power and sample size calculation were not conducted because of the nature of this retrospective health claim data investigation. Statistical significance was set as two-tailed p-values <0.05. SPSS Statistics version 29.0.0 (IBM Corp., Armonk, NY), Python 3.9.0, Pandas 2.0.2, PyCaret 3.1.0, scikit-survival 0.21.0, and Matplotlib 3.5.1 applications were used. The chi-square test, Mann-Whitney U test, Spearman’s correlation coefficient, logistic regression, and Cox regression analysis were performed. The statistical method was the same as that in our previous report [24,25].

Ethical considerations

This study was approved by the Itoigawa General Hospital Ethics Committee (approval number: 2022-2, 2022-10). The requirement for written informed consent was waived due to the anonymous and retrospective nature of the study. All studies adhered to the principles of the Declaration of Helsinki and were carried out following the Strengthening the Reporting of Observational Studies in Epidemiology (STROBE) guidelines.

We herein explain the process and access to the REZULT database. Japan System Techniques Co. Ltd. obtains anonymized health insurance claim data from insurers and sells it as REZULT. Such anonymized data is not considered personal information. Therefore, patient consent is not required for secondary use, as in this study under the Act on the Protection of Personal Information in Japan. In addition, when using anonymized commercial data for medical research, it is not subject to ethical review according to the Japanese Ethical Guideline for Medical Research. However, in this study, an ethical review was performed.

## Results

Study 1 (cross-sectional study): prescription pattern for patients with migraine

Among 3,300,705 patients aged >17 years in the 2020 REZULT database, 66,428 (2.01%) had migraine as a “medical insurance diagnosis” from the health insurance system. Of these migraine patients, musculoskeletal disorders such as back pain were diagnosed in 5474/66,428 (8.24%), psychiatric disorders (e.g., depression and insomnia) in 4198/66,428 (6.32%), and dysmenorrhea in 2803/66,428 (4.22%). The number of patients overprescribed with at least one single NSAID, combination NSAIDs, triptans, or combination of those, and at least one of them were 9280/41,209 (22.52%), 1096 (2.66%), 2118 (5.14%), 4016 (9.74%), and 15,627 (37.92%), respectively (Table 2; Appendices: Table 10).

**Table 2:** Treatment pattern for migraine patients (partially omitted) Full description is available in the Appendices: Table 10 NSAIDs: nonsteroidal anti-inflammatory drugs; SD: standard deviation

Characteristics	Overall	Patients with acute treatment only	Patients with triptan without prophylactic treatment	Patients with both acute and prophylactic treatment	Patients with triptan and prophylactic treatment
Number	41,209 (100.00%)	34,797 (84.44%)	16,891 (40.99%)	6412 (15.56%)	4813 (11.68%)
Number of overprescription (%)	15,627 (37.92%)	12,358 (35.52%)	6530 (38.66%)	3269 (50.98%)	2562 (53.23%)
Single NSAID overprescription, n (%)	9280 (22.52%)	7798 (22.41%)	2598 (15.38%)	1482 (23.11%)	904 (18.78%)
Combination NSAID overprescription, n (%)	1096 (2.66%)	786 (2.26%)	351 (2.08%)	310 (4.83%)	202 (4.20%)
Triptan overprescription, n (%)	2118 (5.14%)	1156 (3.32%)	1156 (6.84%)	962 (15.00%)	962 (19.99%)
Multiple drug types overprescription, n (%)	4016 (9.75%)	3156 (9.07%)	2863 (16.95%)	860 (13.41%)	812 (16.87%)
Age, years, mean ± SD	37.26 ± 11.45	37.43 ± 11.49	36.81 ± 11.08	36.36 ± 11.23	35.79 ± 10.96
Sex: female, n (%)	29,724 (72.13%)	25,119 (72.19%)	12,219 (72.34%)	4605 (71.82%)	3511 (72.95%)
Acute treatment, n (%)	0 (0.00%)	0 (0.00%)			
Combination NSAIDs, n (%)	1029 (2.50%)	880 (2.53%)	-	149 (2.32%)	-
Single NSAIDs, n (%)	17,342 (42.08%)	16,058 (46.15%)	-	1284 (20.02%)	-
Triptan, n (%)	7722 (18.74%)	5988 (17.21%)	5988 (35.45%)	1734 (27.04%)	1734 (36.03%)
Combination and single NSAIDs, n (%)	1133 (2.75%)	866 (2.49%)	-	166 (2.59%)	-
Combination NSAIDs and triptan, n (%)	1206 (2.93%)	967 (2.78%)	866 (5.13%)	340 (5.30%)	340 (7.06%)
Single NSAIDs and triptan, n (%)	11,968 (29.04%)	9504 (27.31%)	9504 (56.27%)	2464 (38.43%)	2464 (51.19%)
All, n (%)	808 (1.96%)	533 (1.53%)	533 (3.16%)	275 (4.29%)	275 (5.71%)
Prophylactic treatment, n (%)	6412 (15.56%)	-	-	6412 (100.00%)	4813 (100.00%)
Single, n (%)	5418 (84.50%)	-	-	5418 (84.50%)	3995 (83.00%)
Two types, n (%)	859 (13.40%)	-	-	859 (13.40%)	697 (14.48%)
More than two types, n (%)	135 (0.21%)	-	-	135 (0.21%)	121 (2.47%)
Calcium-channel blockers (lomerizine), n (%)	2787 (6.76%)	-	-	2787 (43.47%)	2197 (45.65%)
Beta-blockers (propranolol), n (%)	280 (0.68%)	-	-	280 (4.37%)	170 (3.53%)
Anticonvulsants (valproic acid), n (%)	1524 (3.70%)	-	-	1524 (23.77%)	1039 (21.59%)
Antidepressants (amitriptyline), n (%)	642 (1.56%)	-	-	642 (10.01%)	448 (9.31%)
Calcium-channel blockers (verapamil), n (%)	185 (0.45%)	-	-	185 (2.89%)	141 (2.93%)
Lomerizine and beta-blockers, n (%)	51 (0.12%)	-	-	51 (0.80%)	43 (6.79%)
Lomerizine and anticonvulsants, n (%)	397 (0.96%)	-	-	397 (6.19%)	327 (2.66%)
Lomerizine and antidepressants, n (%)	150 (0.36%)	-	-	150 (2.34%)	128 (45.65%)
Beta-blocker and verapamil, n (%)	4 (0.01%)	-	-	4 (0.06%)	2 (2.33%)
Other two combinations, n (%)	257 (0.62%)	-	-	257 (2.00%)	197 (1.24%)
Lomerizine, propranolol, anticonvulsants, n (%)	10 (0.02%)	-	-	10 (0.05%)	10 (2.33%)
Lomerizine, anticonvulsants, antidepressants, n (%)	60 (0.15%)	-	-	60 (0.94%)	55 (1.14%)
Other three combinations, n (%)	54 (0.14%)	-	-	54 (0.79%)	48 (1.14%)
Four or five combinations, n (%)	11 (0.05%)	-	-	11 (0.17%)	8 (0.16%)

Neurologists and neurosurgeons tend to prescribe prophylactic medication more than other departments (Appendices: Table 11). The collection method of the number for these medical departments was not related to specialists certified by the Japan Medical Specialty Board or medical academic societies, or to the Guidelines for the Optimal Use of CGRP-related Drugs in Japan, but was based on the doctors' own prominent medical departments.

Single NSAIDs were prescribed to 31,251/41,209 (75.84%) patients with migraine, and 9280/31,251 (29.70%) were overprescribed with a single NSAID (≥45 tablets/90 days). Among those patients, 1482/9280 (15.97%) received prophylactic treatment. It should be noted that acetaminophen is distributed in 200 mg tablets and that two or three tablets are often used as a single dose (Table 3; Appendices: Table 12).

**Table 3:** Treatment pattern of single NSAID (partially omitted) Full description is available in the Appendices: Table 12 d: days; NSAIDs: nonsteroidal anti-inflammatory drugs; SD: standard deviation; tbl: tablets

Characteristics	Overall	0-11 tbl/90 d	12-29 tbl/90 d	30-44 tbl/90 d	≥45 tbl/90 d
N (%)	31,251 (100.00%)	7361 (23.55%)	9262 (29.64%)	5348 (17.11%)	9280 (29.70%)
Number of tbl/90 d, mean ± SD	47.48 ± 67.2	7.94 ± 2.58	19.45 ± 4.48	36.31 ± 5.08	113.27 ± 93.5
Age, years, mean ± SD	37.4 ± 11.52	36.23 ± 11.53	36.2 ± 11.24	37.03 ± 11.08	39.73 ± 11.67
Sex: female, n (%)	22,631 (72.42%)	4982 (67.68%)	6646 (71.76%)	3970 (74.23%)	7033 (75.79%)
Acute treatment, n (%)					
Single NSAIDs, n (%)	17,342 (55.49%)	3783 (51.39%)	5166 (55.78%)	2963 (55.40%)	5430 (58.51%)
Combination and single NSAIDs, n (%)	1133 (3.63%)	246 (3.34%)	346 (3.74%)	193 (3.61%)	348 (3.75%)
Single NSAIDs and triptan, n (%)	11,968 (38.30%)	3124 (42.44%)	3494 (37.72%)	2067 (38.65%)	3283 (35.38%)
All, n (%)	808 (2.59%)	208 (2.83%)	256 (2.76%)	125 (2.34%)	219 (2.36%)
Prophylactic treatment, n (%)	4189 (13.40%)	850 (11.55%)	1172 (12.65%)	685 (12.81%)	1482 (15.97%)
None, n (%)	27,062 (86.60%)	6511 (88.45%)	8090 (87.35%)	4663 (87.19%)	7798 (84.03%)
Single, n (%)	3513 (11.24%)	753 (10.23%)	997 (10.76%)	585 (10.94%)	1178 (12.69%)
Two types, n (%)	573 (1.83%)	81 (1.10%)	148 (1.60%)	91 (1.70%)	253 (2.73%)
More than two types, n (%)	103 (0.34%)	16 (0.22%)	27 (0.29%)	9 (0.17%)	51 (0.54%)
Calcium-channel blockers (lomerizine), n (%)	1754 (5.61%)	389 (5.28%)	506 (5.46%)	294 (5.50%)	565 (6.09%)
Beta-blockers (propranolol), n (%)	211 (0.68%)	35 (0.48%)	70 (0.76%)	35 (0.65%)	71 (0.77%)
Anticonvulsants (valproic acid), n (%)	1015 (3.25%)	212 (2.88%)	281 (3.03%)	152 (2.84%)	370 (3.99%)
Antidepressants (amitriptyline), n (%)	435 (1.39%)	99 (1.34%)	113 (1.22%)	80 (1.50%)	143 (1.54%)
Calcium-channel blockers (verapamil), n (%)	98 (0.31%)	18 (0.24%)	27 (0.29%)	24 (0.45%)	29 (0.31%)
Lomerizine and anticonvulsants, n (%)	267 (0.85%)	36 (0.49%)	69 (0.74%)	44 (0.82%)	118 (1.27%)
Other two combinations, n (%)	281 (0.91%)	42 (0.57%)	76 (0.81%)	44 (0.82%)	119 (1.27%)
Three combinations, n (%)	93 (0.30%)	16 (0.21%)	25 (0.26%)	9 (0.17%)	43 (0.46%)
Four or five combinations, n (%)	10 (0.04%)	0 (0.00%)	2 (0.02%)	0 (0.00%)	8 (0.08%)

Combination NSAIDs were prescribed to 4176 (10.13%) of 41,209 patients with migraine, and 1096/4176 (26.25%)were overprescribed with combination NSAIDs (≥30 tablets/90 days). Among them, 310/1096 (28.28%) patients received prophylactic treatment (Appendices: Table 13).

Triptans were prescribed to 21,704 of 41,209 (52.67%) patients with migraine (Table 4; Appendices: Table 14), and 2118/21,704 (9.76%) were overprescribed with triptans (≥30 tablets/90 days) with 962/2118 (45.42%) prophylactic treatment.

**Table 4:** Treatment pattern of triptan (partially omitted) Full description is available in Appendices: Table 14 d: days; NSAIDs: nonsteroidal anti-inflammatory drugs; SD: standard deviation; tbl: tablets

Characteristics	Overall	0-11 tbl/90 d	12-29 tbl/90 d	30-44 tbl/90 d	≥45 tbl/90 d
N (%)	21,704 (100.00%)	15,131 (69.72%)	4455 (20.53%)	1414 (6.51%)	704 (3.24%)
Number of tbl/90 d, mean ± SD	12.22 ± 14.12	5.82 ± 2.85	18.36 ± 4.59	34.75 ± 4.82	65.76 ± 28.04
Age, years, mean ± SD	36.58 ± 11.06	36.22 ± 11.14	37.04 ± 10.88	37.72 ± 10.62	39.18 ± 10.83
Sex: female, n (%)	15,730 (72.48%)	10,732 (70.93%)	3404 (76.41%)	1075 (76.03%)	519 (73.72%)
Acute treatment, n (%)					
Triptan, n (%)	7722 (35.58%)	5233 (34.58%)	1633 (36.66%)	552 (39.04%)	304 (43.18%)
Combination NSAIDs and triptan, n (%)	1206 (5.56%)	929 (6.14%)	205 (4.60%)	54 (3.82%)	18 (2.56%)
Single NSAIDs and triptan, n (%)	11,968 (55.14%)	8382 (55.40%)	2449 (54.97%)	752 (53.18%)	385 (54.69%)
All, n (%)	808 (3.72%)	587 (3.88%)	138 (3.10%)	56 (3.96%)	27 (3.84%)
Prophylactic treatment, n (%)	4813 (22.18%)	2409 (15.92%)	1442 (32.37%)	597 (42.22%)	365 (51.85%)
None, n (%)	16,891 (77.82%)	12,722 (84.08%)	3013 (67.63%)	817 (57.78%)	339 (48.15%)
Single, n (%)	3995 (18.41%)	2116 (13.98%)	1167 (26.20%)	456 (32.25%)	256 (36.36%)
Two types, n (%)	697 (3.21%)	265 (1.75%)	224 (5.03%)	123 (8.70%)	85 (12.07%)
More than two types, n (%)	121 (0.56%)	28 (0.18%)	51 (1.15%)	18 (1.27%)	24 (3.41%)
Calcium-channel blockers (lomerizine), n (%)	2197 (10.12%)	1168 (7.72%)	643 (14.43%)	262 (18.53%)	124 (17.61%)
Beta-blockers (propranolol), n (%)	170 (0.78%)	88 (0.58%)	54 (1.21%)	13 (0.92%)	15 (2.13%)
Anticonvulsants (valproic acid), n (%)	1039 (4.79%)	538 (3.56%)	305 (6.85%)	112 (7.92%)	84 (11.93%)
Antidepressants (amitriptyline), n (%)	448 (2.06%)	249 (1.65%)	122 (2.74%)	52 (3.68%)	25 (3.55%)
Calcium-channel blockers (verapamil), n (%)	141 (0.65%)	73 (0.48%)	43 (0.97%)	17 (1.20%)	8 (1.14%)
Lomerizine and beta-blockers, n (%)	43 (0.20%)	19 (0.13%)	10 (0.22%)	6 (0.42%)	8 (1.14%)
Lomerizine and anticonvulsants, n (%)	327 (1.51%)	131 (0.87%)	96 (2.15%)	63 (4.46%)	37 (5.26%)
Lomerizine and antidepressants, n (%)	128 (0.59%)	52 (0.34%)	45 (1.01%)	17 (1.20%)	14 (1.99%)
Anticonvulsants and antidepressants, n (%)	112 (0.52%)	33 (0.22%)	43 (0.97%)	21 (1.49%)	15 (2.13%)
Other two combinations, n (%)	87 (0.40%)	30 (0.19%)	30 (0.68%)	16 (1.12%)	11 (1.55%)
Lomerizine, anticonvulsants, antidepressants, n (%)	55 (0.25%)	12 (0.08%)	25 (0.56%)	4 (0.28%)	14 (1.99%)
Other three combinations, n (%)	58 (0.28%)	14 (0.09%)	23 (0.50%)	13 (0.92%)	8 (1.13%)
Four or five combinations, n (%)	8 (0.03%)	2 (0.02%)	3 (0.07%)	1 (0.07%)	2 (0.28%)

Two or more types of acute medications were prescribed for 15,115/41,209 (36.68%) patients. Any combination of single, combination, and/or triptans, as well as that does not constitute overprescription for a single drug, was overprescribed (≥30 tablets/90 days) to 4016 of 15,115 (26.57%) patients with migraine. Among them, 860/4016 (21.41%) patients received prophylactic treatment (Appendices: Table 15).

We finally investigated the association between prescription patterns, ADI, and the number of headache specialists. There were no missing values concerning regional information. ADI is shown in Figure 1A, and the number of headache specialists per 100,000 people is shown in Figure 1B. ADI and the number of headache specialists were correlated (r=-0.393, p<0.001). The percentage of patients with a migraine prescription out of all REZULT database patients is shown by region in Figure 1C. The percentage of overprescribing and prophylaxis prescribing among all prescriptions for migraine patients are shown in Figures 1D-1E. All the information has been combined in Figure 1F. Logistic regression revealed that older age, female sex, low ADI (not poverty), and lower concentration of headache specialists were related to overprescription. Also, it revealed that younger age and area with higher headache specialists proportion were related to prophylaxis prescription (Table 5).

**Table 5:** Multivariate analysis ADI: areal deprivation index; B: slope

Overprescription	B	Standard error	Wald	P-value	Exp (B)	95% CI lower	95% CI upper
Age (y.o.)	0.02	0.00	555.11	<0.001	1.02	1.02	1.03
Female as 1	0.24	0.03	80.04	<0.001	1.27	1.21	1.34
ADI	-0.12	0.03	16.44	<0.001	0.89	0.83	0.94
The number of headache specialists (/100,000 people)	-0.10	0.05	6.28	0.012	0.89	0.81	0.97
Constant	-1.10	0.30	12.73	<0.001	0.35	-	-
Prophylactic medication	B	Standard error	Wald	p-value	Exp(B)	95%CI lower	upper
Age (y.o.)	-0.01	0.00	34.61	<0.001	0.99	0.99	0.99
Female as 1	-0.02	0.03	0.49	0.486	0.98	0.92	1.04
ADI	-0.05	0.04	1.70	0.192	0.95	0.89	1.02
The number of headache specialists (/100,000 people)	0.23	0.06	15.38	<0.001	1.26	1.12	1.41
Constant	-1.10	0.36	9.32	0.002	0.33	-	-

Study 2 (longitudinal study): overprescription during the study period

**Figure 2:**
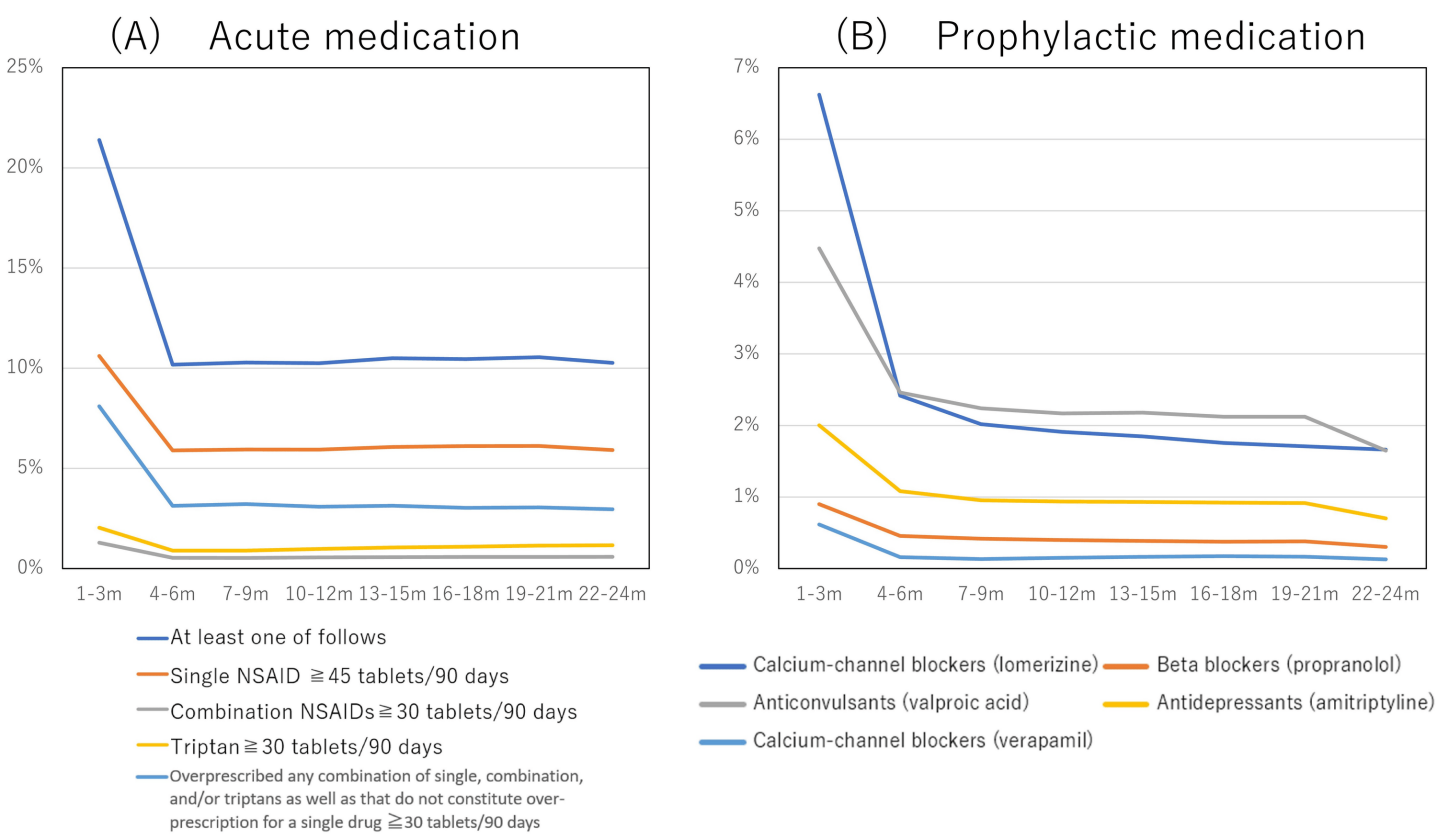
Prescription pattern during the two years from the initial diagnosis (A) Percentage of overprescriptions with acute medications in each term. (B) Percentage of prescriptions for prophylactic medication in each term. The number of patients with prescription were 296,164(1-3 m), 280,747 (4-6 m), 266,742 (7-9 m), 253,358 (10-12 m), 241,497 (13-15 m), 230,567 (16-18 m), 219,273 (19-21 m), and 219,272 (22-24 m), respectively m; months; NSAID: nonsteroidal anti-inflammatory drug

Among 6,840,618 patients from the REZULT database, 296,164 (4.33%) were first diagnosed with migraine in the health insurance system, and this insurance disease name was regularly found for at least two consecutive years during the studied period (July 2010 to April 2022). Of these migraine patients, musculoskeletal disorders such as back pain were diagnosed in 24,611/296,164 (8.31%), psychiatric disorders in 18,481/296,164 (6.24%), and dysmenorrhea in 1324/296164 (4.47%). In Japan, prescriptions can be given for up to 90 days. Among patients with two or more continuous prescriptions, the intervals between prescriptions are as follows: one to two weeks (36.55%), three to four weeks (11.68%), five to six weeks (9.81%), seven to nine weeks (23.63%), 10-11 weeks (9.12%), and over 12 weeks (9.20%). During the first 90 days after migraine diagnosis, 31,421/296,164 (10.61%) patients received >45 tablets of a single NSAID, 3834/296,164 (1.29%) received >30 tablets of combination NSAIDs, and 6028/296,164 (2.03%) received >30 tablets of triptans. In the first three months, both overprescribing of acute medications and prescribing of prophylactic medications were observed in large numbers; after four to six months and up to 21-24 months, their proportions remained almost unchanged (Figure 2A; Table 6).

**Table 6:** Changes in prescribing patterns over time "n" indicates the number of patients with prescriptions in each month d: days; NSAIDs: nonsteroidal anti-inflammatory drugs; tbl: tablets

Variables	1-3 months (n=296,164), n (%)	4-6 months (n=280,747), n (%)	7-9 months (n=266,742), n (%)	10-12 months (n=253,358), n (%)	13-15 months (n=241,497), n (%)	16-18 months (n=230,567), n (%)	19-21 months (n=219,273), n (%)	22-24 months (n=219,272), n (%)
At least one of the following	63,349 (21.39%)	28,580 (10.18%)	27,421 (10.28%)	25,969 (10.25%)	25,357 (10.50%)	24,117 (10.46%)	23,133 (10.55%)	22,519 (10.27%)
Single NSAID ≥45 tbl/90 d	31,423 (10.61%)	16,564 (5.90%)	15,844 (5.94%)	15,049 (5.94%)	14,659 (6.07%)	14,088 (6.11%)	13,420 (6.12%)	12,981 (5.92%)
Combination NSAIDs ≥30 tbl/90 d	3821 (1.29%)	1516 (0.54%)	1414 (0.53%)	1419 (0.56%)	1352 (0.56%)	1337 (0.58%)	1272 (0.58%)	1294 (0.59%)
Triptan ≥30 tbl/90 d	6042 (2.04%)	2527 (0.90%)	2401 (0.90%)	2483 (0.98%)	2536 (1.05%)	2513 (1.09%)	2522 (1.15%)	2544 (1.16%)
Overprescribed any combination of single, combination, and/or triptans as well as that does not constitute overprescription for a single drug	23,989 (8.10%)	8787 (3.13%)	8589 (3.22%)	7829 (3.09%)	7583 (3.14%)	6986 (3.03%)	6688 (3.05%)	6490 (2.96%)
Prophylactic treatment	37,879 (12.79%)	15,862 (5.65%)	13,204 (4.95%)	12,060 (4.76%)	11,350 (4.70%)	10,445 (4.53%)	9802 (4.47%)	9626 (4.39%)
Calcium-channel blockers (lomerizine)	19,606 (6.62%)	6794 (2.42%)	5388 (2.02%)	4839 (1.91%)	4468 (1.85%)	4058 (1.76%)	3771 (1.72%)	3640 (1.66%)
Beta-blockers (propranolol)	2665 (0.90%)	1263 (0.45%)	1120 (0.42%)	1013 (0.40%)	918 (0.38%)	853 (0.37%)	833 (0.38%)	855 (0.39%)
Anticonvulsants (valproic acid)	13,268 (4.48%)	6906 (2.46%)	5975 (2.24%)	5498 (2.17%)	5265 (2.18%)	4888 (2.12%)	4649 (2.12%)	4627 (2.11%)
Antidepressants (amitriptyline)	5923 (2.00%)	3032 (1.08%)	2534 (0.95%)	2382 (0.94%)	2246 (0.93%)	2121 (0.92%)	2017 (0.92%)	1952 (0.89%)
Calcium-channel blockers (verapamil)	1836 (0.62%)	449 (0.16%)	347 (0.13%)	380 (0.15%)	386 (0.16%)	392 (0.17%)	351 (0.16%)	351 (0.16%)
1 type	33,022 (11.15%)	13,560 (4.83%)	11,257 (4.22%)	10,236 (4.04%)	9587 (3.97%)	8785 (3.81%)	8201 (3.74%)	8025 (3.66%)
2 types	4354 (1.47%)	2021 (0.72%)	1707 (0.64%)	1596 (0.63%)	1521 (0.63%)	1476 (0.64%)	1425 (0.65%)	1425 (0.65%)
3 types	474 (0.16%)	309 (0.11%)	240 (0.09%)	203 (0.08%)	217 (0.09%)	184 (0.08%)	175 (0.08%)	175 (0.08%)
4 types	30 (0.01%)	0 (0.00%)	0 (0.00%)	0 (0.00%)	24 (0.01%)	0 (0.00%)	0 (0.00%)	0 (0.00%)
5 types	0 (0.00%)	0 (0.00%)	0 (0.00%)	0 (0.00%)	0 (0.00%)	0 (0.00%)	0 (0.00%)	0 (0.00%)

**Figure 3:**
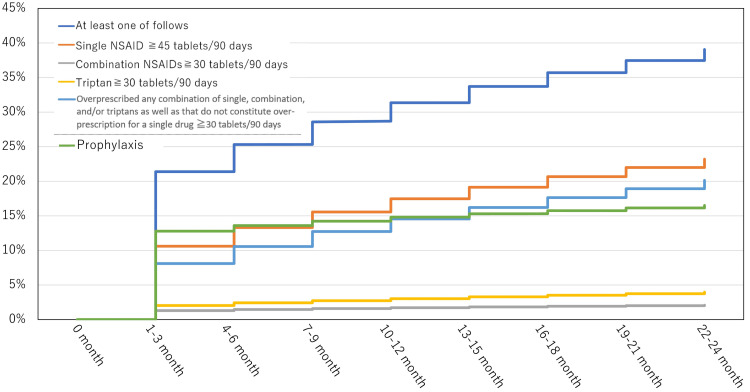
Kaplan-Meier curves for overprescription or prophylactic medication during the two years from the initial diagnosis (A) Percentage of overprescriptions of acute medications: two years after the initial visit, the proportion of patients receiving overprescribed acute medications (≥30 or 45 tablets/90 days) gradually declined. Over the two-year period, 115,361 out of 296,164 patients (38.95%) were overprescribed acute medications within a 90-day period at least once. (B) Percentage of prophylactic medication prescriptions: throughout the two-year period, 48,886 of the 296,164 patients (16.51%) received prophylactic medication at least once X-axis: time; Y-axis: proportion of overprescription or prophylactic medication prescription NSAID; nonsteroidal anti-inflammatory drug

Throughout the two years, 115,361/296,164 (38.95%) patients were overprescribed acute medications in 90 days at least once. Subanalysis revealed that the number of patients overprescribed with a single NSAID (≥45 tablets/90 days), combination NSAIDs, triptans, or any combination of single, combination, and/or triptans as well as those that do not constitute overprescription for a single drug (≥30 tablets/90 days) was 68,704/296,164 (23.20%), 6173/296,164 (2.08%), 11,755/296,164 (3.97%), and 59,635/296,164 (20.14%), respectively (Figure 3).

Then, 35,954/296,164 (12.14%) patients continued to have overprescription for two consecutive terms (four to six months), 10,721/296,164 (3.62%) patients for four consecutive terms (10-12 months), and 2340/296,164 (0.79%) patients for eight consecutive terms (22-24 months). Prophylactic medication was initiated in the first 90 days in 37,877/296,164 (12.79%) patients, which decreased to 9634/296,164 (3.25%) patients after two years (Table 6; Figure 2B). Throughout the two years, 48,886/296,164 (16.51%) patients with migraines were prescribed prophylactic medications on at least one occasion (Figure 3). Then, 18,629/296,164 (6.29%) patients continued to have prophylactic medication for two consecutive terms (four to six months), 773/296,164 (2.61%) patients for four consecutive terms (10-12 months), and 3200/296,164 (1.08%) patients for eight consecutive terms (22-24 months) (Table 7).

**Table 7:** Overprescription and prophylaxis prescription duration (n=296,164) ^*^The same drug’s overprescription is not always consecutive. The pattern includes triptan overprescription in one term, single NSAID overprescription in the next term, and so on NSAID; nonsteroidal anti-inflammatory drug

Overprescription	Any 1 term (1-3 months), n (%)	Any 2 or more continuous terms (4-6 continuous months), n (%)	Any 3 or more continuous terms (7-9 continuous months), n (%)	Any 4 or more continuous terms (10-12 continuous months), n (%)	8 continuous terms (21-24 continuous months), n (%)
At least one of the following^*^	115,356 (38.95%)	35,954 (12.14%)	17,770 (6.00%)	10,721 (3.62%)	2340 (0.79%)
Single NSAID ≥45 tbl/90 d	68,710 (23.20%)	19,961 (6.74%)	9714 (3.28%)	5805 (1.96%)	1274 (0.43%)
Combination NSAIDs ≥30 tbl/90 d	6160 (2.08%)	2103 (0.71%)	1244 (0.42%)	800 (0.27%)	178 (0.06%)
Triptan ≥30 tbl/90 d	11,758 (3.97%)	3672 (1.24%)	1866 (0.63%)	1096 (0.37%)	148 (0.05%)
Overprescribed any combination of single, combination, and/or triptans as well as that does not constitute overprescription for a single drug	59,647 (20.14%)	5538 (1.87%)	888 (0.30%)	207 (0.07%)	0 (0.00%)
Prophylactic treatment	48,897 (16.51%)	18,629 (6.29%)	11,402 (3.85%)	7730 (2.61%)	3199 (1.08%)

**Figure 4:**
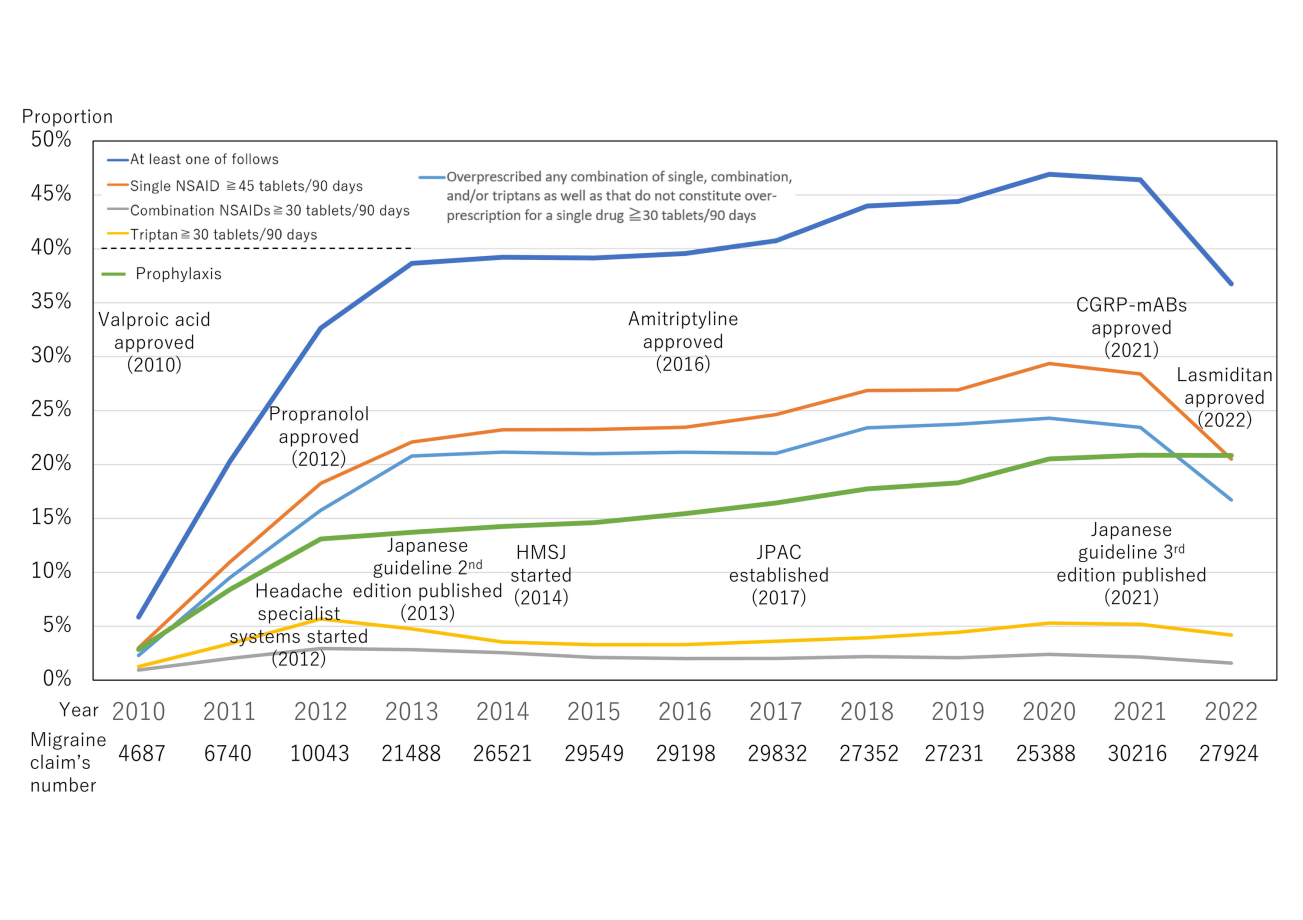
Prescribing pattern from 2010 to 2022 With the advent of calcitonin gene-related peptide-related monoclonal antibodies (CGRP-mABs) in 2021, overprescription decreased, and prophylactic treatment prescribing increased HMSJ: headache master school in Japan; JPAC; Japan Patient Advocacy Coalition; NSAID: nonsteroidal anti-inflammatory drug

Figure 4 shows the prescribing pattern from 2010 to 2022, indicating that overprescription decreased and prophylactic treatment prescribing increased, with the advent of CGRP-related monoclonal antibodies and the publication of the new guideline.

Log-minus-log plots confirmed the proportional hazard assumption, and there were no variables with multicollinearity confirmed by variance inflation factors. Cox regression analysis (n=217,246 with areal information) revealed that older age, more prescriptions of single and combination NSAIDs, and triptans in the first three months, and prescription of prophylactic medication during the first three months (p<0.001, all), and a small number of headache specialists (p=0.002) were risk factors for overprescription in the two years. Interestingly, high ADI (poverty) and a small number of headache specialists were associated with the overprescription of a single NSAID. Low ADI (not poverty) was related to overprescription of combination NSAIDs and triptan and prophylaxis prescription. The large number of headache specialists tended to increase the prescription of prophylaxis, but this was not related to triptan overprescription in 90 days (Table 8; Appendices: Table 16).

**Table 8:** Cox regression analyses results for 90-day overprescription (one-term) (n=217,246) (partially omitted) Full description is available in the Appendices: Table 16 ADI: areal deprivation index; B: slope; d: days; NSAIDs: nonsteroidal anti-inflammatory drugs; tbl: tablets

Overprescription of any type of medication	B	Standard error	Wald	P-value	Odds ratio	95% CI lower	95% CI upper
Female	0.16	0.01	450.55	<0.001	1.17	1.15	1.18
Age (y.o.)	0.01	0.00	2034.56	<0.001	1.01	1.01	1.01
Single NSAID during the first 90 d (tbl/90 d)	0.00	0.00	207.60	<0.001	1.00	1.00	1.00
Combination NSAIDs during the first 90 d (tbl/90 d)	0.00	0.00	134.03	<0.001	1.00	1.00	1.00
Triptan during the first 90 d (tbl/90 d)	0.00	0.00	1130.40	<0.001	1.00	1.00	1.00
Presence of prophylactic treatment prescription during the first 90 d	0.26	0.01	953.97	<0.001	1.30	1.28	1.32
ADI	0.01	0.01	1.21	0.271	1.01	0.99	1.02
The number of headache specialists (/100,000 people)	-0.04	0.01	9.50	0.002	0.97	0.94	0.99
Single NSAID	B	Standard error	Wald	P-value	Odds ratio	95% CI lower	95% CI upper
Female	0.17	0.01	332.38	<0.001	1.19	1.17	1.21
Age (y.o.)	0.019	0.00	3131.61	<0.001	1.02	1.02	1.02
Single NSAID during the first 90 d (tbl/90 d)	0.00	0.00	376.71	<0.001	1.00	1.00	1.00
Combination NSAIDs during the first 90 d (tbl/90 d)	0.00	0.00	16.91	<0.001	1.00	1.00	1.00
Triptan during the first 90 d (tbl/90 d)	0.00	0.00	2.52	0.113	1.00	1.00	1.00
Presence of prophylactic treatment prescription during the first 90 d	0.152	0.11	178.15	<0.001	1.16	1.14	1.12
ADI	0.084	0.01	76.20	<0.001	1.09	1.07	1.11
The number of headache specialists (/100,000 people)	-0.06	0.02	13.32	<0.001	0.95	0.92	0.98
Triptan	B	Standard error	Wald	P-value	Odds ratio	95% CI lower	95% CI upper
Female	0.11	0.02	22.01	<0.001	1.12	1.07	1.17
Age (y.o.)	0.01	0.00	90.77	<0.001	1.01	1.01	1.01
Single NSAID during the first 90 d (tbl/90 d)	0.00	0.00	1.58	0.21	1.00	1.00	1.00
Combination NSAIDs during the first 90 d (tbl/90 d)	0.00	0.00	3.36	0.07	1.00	1.00	1.00
Triptan during the first 90 d (tbl/90 d)	0.00	0.00	3383.14	<0.001	1.00	1.00	1.00
Presence of prophylactic treatment prescription during the first 90 d	1.34	0.02	3996.90	<0.001	3.81	3.66	3.97
ADI	-0.12	0.03	23.68	<0.001	0.88	0.84	0.93
The number of headache specialists (/100,000 people)	0.01	0.04	0.04	0.84	1.01	0.94	1.08
Prophylactic medication	B	Standard error	Wald	P-value	Odds ratio	95% CI lower	95% CI upper
Female	-0.06	0.01	30.84	<0.001	0.94	0.92	0.96
Age (y.o.)	0.00	0.00	110.86	<0.001	1.00	0.99	1.00
Single NSAID during the first 90 d (tbl/90 d)	0.00	0.00	0.04	0.837	1.00	1.00	1.00
Combination NSAIDs during the first 90 d (tbl/90 d)	0.00	0.00	9.51	0.002	1.00	1.00	1.00
Triptan during the first 90 d (tbl/90 d)	0.00	0.00	1659.95	< .001>	1.00	1.00	1.00
ADI	-0.05	0.01	16.49	< .001>	0.95	0.93	0.97
The number of headache specialists (/100,000 people)	0.23	0.02	149.13	< .001>	1.26	1.21	1.30

These trends were similar even when the objective variable was 180 days (two terms) of overprescribing instead of 90 days (one term) of overprescribing (Appendices: Table 17).

## Discussion

In the cross-sectional study, among 41,209 patients diagnosed with migraine and treated with prescribed medication in 2020, only 6412/41,209 (15.56%) received prophylactic medication. The number of patients overprescribed with single NSAID or triptans was 9280/41,209 (22.52%) and 2118/41,209 (5.14%), respectively. Multivariate analysis revealed that older age, female sex, low ADI (not poverty), and lower concentration of headache specialists were related to overprescription and that younger age and area with higher headache specialists proportion were related to prophylaxis prescription. In the longitudinal study, throughout these two years from initial migraine diagnosis, a single NSAID or triptans overprescription was found in 68,704/296,164 (23.20%), and 11,755/296,164 (3.97%), respectively, and 48,886/296,164 (16.51%) patients were prescribed prophylactic medications on at least one occasion.

No increase in the rate of overprescription was confirmed over two years. Early prescription of prophylactic medication was related to all types of overprescription. Hence, it can be regarded that patients’ migraine was also so severe that they had frequent headache days at the initial consultation, requiring a high number of tablets of acute medications and early treatment with prophylactic medications. High ADI (poverty) and lower concentration of headache specialists were associated with the overprescription of a single NSAID. Low ADI (not poverty) was related to overprescription of combination NSAIDs and triptan and prophylaxis prescription, and a large number of headache specialists tended to increase prophylaxis prescription. Based on the results of these two studies, raising awareness of the relationship between overprescription and the values of ADI, the number of headache specialists, and patients with early prescriptions of prophylactic medication may help reduce the rate of overprescription.

Investigation of foreign health insurance claims databases

Here, we discuss recent findings concerning the overprescription of triptans. This is because, unlike NSAIDs, triptans require a migraine diagnosis for the prescription. A German study in 2005 revealed that triptans were used by 85,172 (1.3%) of the 6.7 million population studied, and 8,844/85,172 (10.4%) of these were suspected overusers [14]. A French study in 2010 and 2011 revealed that 2243 of 99,450 triptan users (2.3%) could be overusers. Older age and the use of prophylactic medications, benzodiazepines, and antidepressants were risk factors for triptan overuse [19]. An Italian study, which investigated two areas in 2012, showed that approximately 10% of triptan users were frequent users (≥10 tablets/month), and two-thirds of them maintained their frequent triptan use for an additional three months [18]. An Austrian research in 2018 found that 33,062/5,918,487 (0.56%) of the population were triptan users, and 1970/33,062 (6.0%) were overusers. Overusers were three years older than non-overusers [16].

Similar to these reports worldwide, our results exhibited the same trends. Triptans were prescribed to 21,704 of 41,209 (52.67%) individuals with migraine, and those comprised 0.66% (21,704/3,300,705) of the whole studied population; 2118/21,704 (9.76%) were suspected overusers. To improve the overprescription of triptan, raising awareness of the appropriate triptan use and prevention of overuse/overprescription are needed worldwide.

Previous Japanese studies using health insurance claims data

Meyers et al. [20] investigated patients with migraines and their treatment patterns using data from the Japan Medical Data Centre (JMDC) database. In three years (May 1, 2011, to April 30, 2014), among 16,433 patients with migraines, 9873 (60.1%) received only acute medication, 3022 (18.4%) received prophylactic medications, and 3548 (21.6%) were not prescribed any medication. After 61.2 ± 65.3 days of initial prophylactic treatment, 592/882 (62.2%) of patients discontinued it, whose index migraine treatment regimen was prophylactic treatment only; among them, 105/592 (17.7%) reinitiated the initial treatment, and 44/592 (7.4%) switched the treatment regimen within one year. Similar to Meyer et al.’s [20] findings, in our study, the rate of prescriptions for preventive medications decreased at four to six months (Figure 2 and Table 6), and continuous use of prophylactic medication was not frequently observed (Table 7). Most patients may discontinue treatment after about three months of visits, and only about 10-20% may receive ongoing preventive treatment. Further awareness of the continuation of preventive treatment is needed.

Takizawa et al. [23] investigated 165,339 patients’ datasets in the JMDC database from July 2017 to July 20, revealing that 34,309/165,339 (20.8%) received prophylactic treatment. Of the patients potentially not managed well by acute/preventive treatment, 4229/114,931 (3.7%) had the risk of MOH. This trend was similar to ours, but it should be noted that the exclusion of those who have not been followed up for treatment for more than one year differs from our criteria. Sakai et al. [21] investigated anonymized online survey data coupled with medical claims data (JMDC) of individuals aged 19-74 years from December 1, 2017, to November 30, 2020. Migraine prevalence was 3.2% (691/21,480) and was highest among 30-39-year-olds. Among them, 81.0% (560/691) of patients did not consult a doctor, and 57.8% (362/691) used OTC drugs only. However, only 6.1% (38/691) of patients used prescription medicines. They concluded that findings of an epidemiological study conducted 20 years ago [27] and another study [21] suggested that even with the advent of triptans and CGRP-related drugs and the paradigm shift that has occurred, a lack of appropriate consultation behavior and use of prophylactic treatment persist [28]. Similar trends were confirmed in another study [22].

However, we have shown that after the introduction of new drugs and new guidelines, overprescription was decreasing, and prophylactic prescribing was increasing in 2022 (Figure 4). Whether this trend is due to sampling bias remains to be seen; however, we have certainly made progress toward eliminating the unmet medical need for migraine. Further observation over time is warranted.

Timing of initial consultation and migraine progression

In general, prophylactic medications aim to reduce headache frequency and severity as well as minimize the need for acute care medications [1]. However, our study revealed that those receiving prophylactic treatment within the first 90 days tended to have overprescribed acute medications than those without prophylaxis. This suggests that patients requiring two-year long-term follow-up and early prophylactic use may have consulted doctors for the first time after their migraines had progressed significantly.

Migraine is considered a progressive condition, with episodic cases potentially evolving into chronic migraine at a rate of 2.5% per year. Factors such as high attack frequency, MO, comorbid pain syndromes, and obesity contribute to migraine progression [29]. Our findings align with this, showing a risk of early overprescription of acute medications within the initial three months.

Hirata et al. [22] found that 14-38% of patients with headaches did not visit a hospital because OTC medicines are effective. In contrast, some patients first visited a doctor because OTC had become ineffective (48/691, 6.9%) or their headaches had increased in frequency (55/691, 8.0%). Prophylactic treatment can be terminated relatively early after migraine remission [1,2,21] and is not well-performed in Japan [30,31]. A similar trend is suspected in other countries as well; a study showed that only one in three migraine patients take acute prescribed medications for a migraine attack, and less than one in five patients take prophylactic prescribed medications [10]. Additionally, misdiagnosis and delayed diagnosis of chronic migraine are problematic [32].

We propose that patients receiving early prophylactic treatment may already be in the early stages of developing chronic migraines. To address this, increased educational efforts are needed [5,33], encouraging physicians to treat patients eligible for prophylactic treatment and patients to consult doctors during the early stages of episodic migraines. Also, the number of headache specialists should be raised.

ADI and prescription pattern

The consideration that the distribution of knowledge about a disease varies according to the level of poverty in the community has been examined in the context of other diseases [11,12]. Areal socioeconomic deprivation indices (e.g., ADI) are closely associated with non-economic factors, including insufficient material resource allocations, weak social relationships, and poor self-assessment, which negatively affect health [34]. They also relate to accessibility for medication services and their quality [35]. Rates of appropriate medical consultation and treatment are low among migraineurs [30], and the idea that these external factors including ADI influence them is reasonable.

High ADI was related to overprescription of single NSAIDs. The percentage of the elderly is included in the ADI calculation. Therefore, there are many old people in areas with high ADI, and the community as a whole may tend to avoid triptan and prescribe single NSAIDs because of side effects. It is also possible that fewer doctors and patients use triptans and preventive treatments because fewer doctors and patients can obtain correct disease information, being caught in the migraine stigma [5].

Low ADI was related to the presence of prophylaxis prescriptions. More intelligent and high-society individuals with migraine tend to consult doctors [36]. The lower the ADI, the greater the number of specialists. Both patients and doctors likely have access to the right information about migraine. On the other hand, low ADI is also related to overprescription of combination NSAIDs and triptans. The use of stronger drugs such as triptan for more appropriate acute treatment of migraine is widespread, but the risk of overuse may not yet be widely recognized. The first prevalence survey of MOH was conducted recently in Japan [37], and it is still not widely known even in areas with small ADI.

In this study, we focused only on prescribing patterns obtained from the claim database. The real reason why ADIs affect treatment patterns needs further investigation. However, this study suggests that ADIs can be used as an indicator for raising awareness about overprescription, specific to each group of acute medications. We would also like to examine regional differences in migraine characteristics, treatment outcomes, and comorbidities [38].

Headache specialists

Our findings revealed that the areas with a higher proportion of headache specialists tend to prescribe prophylactic medications and avoid overprescription. Since the establishment of the headache specialist system in Japan in 2012, there have been approximately 1,000 headache specialists, and this number is gradually on the rise. Becoming a headache specialist requires training and examination, and the specialists are expected to provide specialized care that includes correct diagnosis and guideline-based preventive treatment. In addition, headache specialists are expected to provide local leadership in educating the community about local headache care. In the present study, our data showed that appropriate prescribing was more prevalent in areas with more headache specialists. The efforts of the Japanese Headache Society to date have been analyzed, and it is hoped that the number of headache specialists will increase and that they will play an even more active role in the future.

Limitations

This study had several limitations. Firstly, there was a sampling bias, and overestimation cannot be ruled out. We chose the 90-day period with the highest number of acute medications prescribed. Since it was not possible to determine the actual number of days acute medications were used during the study period, we counted the "number of tablets per 90 days" rather than the "number of days per month" of usage. While this method follows an established approach [14], accurate recordings using headache diaries or other methods are more desirable. Secondly, NSAIDs were prescribed not only for migraines but also for other chronic pain conditions, such as back pain, making it difficult to exclude patients using analgesics regularly for other reasons, potentially leading to overestimation. In Japan, migraines are often diagnosed and treated by general practitioners rather than neurologists [39]. Additionally, some patients have coexisting musculoskeletal disorders (5,474/66,428, or 8.24%) or dysmenorrhea (2,803/66,428, or 4.22%), contributing to potential NSAID overestimation. However, our results are still significant regarding triptan prescriptions, which are specific to migraines.

Third, the study involved a retrospective analysis of administrative claims data. Patients were identified solely based on available database information, meaning we could not assess migraine severity, specific characteristics, the actual number of medications taken daily, or the use of OTC medicines. Since some migraine treatments also serve other purposes (e.g., anticonvulsants and antidepressants), it is possible that some patients were mistakenly classified as receiving migraine treatment when, in fact, it was for different comorbidity. Fourth, patients who used insurance systems not captured in the REZULT database could not be tracked, leading to potential regional bias. Fifth, it was challenging to distinguish between patients with "headaches" and those with "migraine," as some diagnoses were labeled for insurance claims but may not have been updated or removed. Additionally, a previous insurance-based study found that 21.6% of migraine patients were not prescribed medications [20], and such patients could not be evaluated. Sixth, it is undeniable that many cases of MOH classified under ICHD-3 code 8.2.6 (MOH attributed to multiple drug classes not individually overused), may be overestimated due to the way the number of tablets is counted. Seventh, we performed ADI calculation by prefecture, but a smaller area needs to be studied more.

Eighth, the analysis took a relatively macro perspective, tracking prescribing patterns every three months; it cannot be ruled out that some people may appear to be overprescribing when cut out in three-month increments due to prescribing every one month, every two months, etc. In the future, analysis from a micro perspective, such as monthly or weekly prescribing patterns, should also be considered. Ninth, one term could be overvalued about overprescription. We also calculated overprescription for consecutive two, four, or eight terms, and it should be noted that two terms may actually be a credible chronic overprescription. Moving forward, we will continue to monitor how prescribing patterns evolve with increased headache awareness [5] and the introduction of new treatments, such as CGRP-targeting medications and lasmiditan. Also, if there is a health insurance claim with overprescription of NSAIDs or triptans, the doctors and patients are not necessarily informed of the incorrect prescription. We are now developing a system to inform them of incorrect prescriptions and reduce overprescription.

## Conclusions

We utilized REZULT, a large health insurance database, to examine migraine prescription patterns. Our results revealed that overprescription of acute medication occurred at a moderate to high rate, and NSAIDs, in particular, are overprescribed at a rate of almost one out of four. Thus, doctors need to be more careful not to casually prescribe NSAIDs. As per real-world data, early prescription of prophylactic medication is related to all types of overprescription, but we surmised that this data does not mean that prophylaxis itself causes overprescribing. Doctors should keep in mind that patients who are immediately prescribed prophylactic medication at their first visit may have a more severe clinical presentation, and in these patients, overprescription is common in their early stages of treatment. Regional socioeconomic status and the number of headache specialists were associated with the overprescription and prophylaxis prescription. These results suggest that appropriate therapeutic intervention by headache specialists reduces the rate of overprescription and enables early intervention for prophylactic medication.

## Appendices

**Table 9:** Drug codes NSAID: nonsteroidal anti-inflammatory drug

Single NSAID	Combination NSAIDs	Triptan	Lomerizine	Propranolol	Valproic acid	Amitriptyline	Verapamil
620007150	621499701	610462006	610432015	620252713	620007035	611170810	620004629
621392002	621499601	622563201		620252707	620065901	611170801	620309102
620100901	621558101	622545001		620252712	622050901	620134702	620007085
620099003		622562501		620293401	620065201	620134603	622015601
620005142		622563203		612120017	621748202	611170474	622892100
621534501		622541301		612120016	622012303	611170473	
620007152		620000419		620004453	621390601		
620000184		620000420			622042801		
621215101		622794201			610412139		
620007151		622425801			621210704		
622229501		622436401			620066601		
622229601		622417301			620004028		
622232501		622413801			622276201		
622242801		622410701			620065401		
622252201		622418601			620065501		
622229701		622417303			622030102		
622325600		622418101			621390701		
620099602		610451019			621748201		
621215602		610462009			622012302		
620100001		622208101			620066301		
621215301		622223201			620065701		
620099201		622194401			620000073		
620099601		622189601			620065001		
621215401		622201001			611130032		
620099301		622518001			620003578		
620100501		622187401			620003577		
620099701		622223204			620006033		
620098902		622221401			620066201		
620100602		622864401			611130089		
620100702		622204501			620066101		
620099101		622211601			611130088		
620100401		622213501			620003156		
620099501		622211101			620003976		
620098501		622658401			620002054		
621808201		622652001			622708700		
620098702		622834401			622708800		
620098801		622657001			622708900		
620098401		622661301			622709000		
620007096		622647901			622709100		
620008628		622650501			622845100		
622709500		622650001			622845200		
622314000		622663101			622887900		
620079349		660470003					
620079315		610451012					
620079325		620006199					
620079305		620006771					
620079303		622189701					
620079338		622846400					
620079311		622846500					
620079345		622846600					
621212601		640443001					
620080001							
620005975							
622381201							
620000033							
620002023							
620000032							
620002022							
620067311							
620002510							
622649001							
622556901							
621677503							
621522602							
621634101							
622556801							
621683101							
621522601							
621677401							
621676901							
620002636							
621520803							
620001953							
620002634							
621526501							
620002632							
620071204							
620002633							
620008279							
621795801							
621795901							
621520701							
621634201							
611140784							
620067313							
620067312							
620000476							
611140022							
620007095							
621968301							
622440201							
661140181							
661140180							
622802501							
622259801							
620007813							
622259401							
622251001							
622260201							
620007812							
622237301							
622252901							
622252301							
622260301							
620004626							
622259301							
622000401							
622250302							
622250901							
622262201							
622261401							
620003477							
622261501							
622817001							
622232601							
622250202							
622259701							
622235401							
622261301							
622259601							
622261801							
622252801							
622237201							
622242901							
622235501							
622257001							
622262001							
622240501							
622234101							
622252401							
622240601							
622262101							
622234201							
622247901							
622227401							
622256901							
622259501							
622240502							
622235301							
621215601							
622237101							
622227301							
622240602							
622251101							
622232701							
622234301							
622238701							
622261901							
622257201							
622261601							
622407001							
622247801							
622363301							
622257101							
622240701							
622227201							
622238801							
620008625							
622363201							
622250301							
622240702							
622507601							
622263701							
622250201							
622253401							
622253301							
622424001							
620072001							
621795701							
622060902							
622060802							
620072501							
622060801							
621557001							
621556901							
622054401							
622060901							
621391101							
621938202							
621953701							
622054402							
621391001							
620002631							

**Table 10:** Treatment pattern for migraine patients (expansion of Table 2) NSAID: nonsteroidal anti-inflammatory drug; SD: standard deviation

Characteristics	Overall	Patients with acute treatment only	Patients with triptan without prophylactic treatment	Patients with both acute and prophylactic treatment	Patients with triptan and prophylactic treatment
N (%)	41,209 (100.00%)	34,797 (84.44%)	16,891 (40.99%)	6412 (15.56%)	4813 (11.68%)
Number of overprescription (%)	15,627 (37.92%)	12,358 (35.52%)	6530 (38.66%)	3269 (50.98%)	2562 (53.23%)
Single NSAID overprescription, n (%)	9280 (22.52%)	7798 (22.41%)	2598 (15.38%)	1482 (23.11%)	904 (18.78%)
Combination NSAID overprescription, n (%)	1096 (2.66%)	786 (2.26%)	351 (2.08%)	310 (4.83%)	202 (4.20%)
Triptan overprescription, n (%)	2118 (5.14%)	1156 (3.32%)	1156 (6.84%)	962 (15.00%)	962 (19.99%)
Multiple drug type overprescription, n (%)	4016 (9.75%)	3156 (9.07%)	2863 (16.95%)	860 (13.41%)	812 (16.87%)
Age, years, mean ± SD	37.26 ± 11.45	37.43 ± 11.49	36.81 ± 11.08	36.36 ± 11.23	35.79 ± 10.96
Sex: female, n (%)	29,724 (72.13%)	25,119 (72.19%)	12,219 (72.34%)	4605 (71.82%)	3511 (72.95%)
Acute treatment, n (%)					
Combination NSAIDs, n (%)	1029 (2.50%)	880 (2.53%)	-	149 (2.32%)	-
Single NSAIDs, n (%)	17,342 (42.08%)	16,058 (46.15%)	-	1284 (20.02%)	-
Triptan, n (%)	7722 (18.74%)	5988 (17.21%)	5988 (35.45%)	1734 (27.04%)	1734 (36.03%)
Combination and single NSAIDs, n (%)	1133 (2.75%)	866 (2.49%)	-	166 (2.59%)	-
Combination NSAIDs and triptan, n (%)	1206 (2.93%)	967 (2.78%)	866 (5.13%)	340 (5.30%)	340 (7.06%)
Single NSAIDs and triptan, n (%)	11,968 (29.04%)	9504 (27.31%)	9504 (56.27%)	2464 (38.43%)	2464 (51.19%)
All, n (%)	808 (1.96%)	533 (1.53%)	533 (3.16%)	275 (4.29%)	275 (5.71%)
Prophylactic treatment, n (%)	6412 (15.56%)	-	-	6412 (100.00%)	4813 (100.00%)
Single, n (%)	5418 (84.50%)	-	-	5418 (84.50%)	3995 (83.00%)
Two types, n (%)	859 (13.40%)	-	-	859 (13.40%)	697 (14.48%)
Three types, n (%)	124 (1.93%)	-	-	124 (1.93%)	113 (2.35%)
Four types, n (%)	9 (0.14%)	-	-	9 (0.14%)	6 (0.12%)
Five types, n (%)	2 (0.03%)	-	-	2 (0.03%)	2 (0.04%)
Calcium-channel blockers (lomerizine), n (%)	2787 (6.76%)	-	-	2787 (43.47%)	2197 (45.65%)
Beta-blockers (propranolol), n (%)	280 (0.68%)	-	-	280 (4.37%)	170 (3.53%)
Anticonvulsants (valproic acid), n (%)	1524 (3.70%)	-	-	1524 (23.77%)	1039 (21.59%)
Antidepressants (amitriptyline), n (%)	642 (1.56%)	-	-	642 (10.01%)	448 (9.31%)
Calcium-channel blockers (verapamil, n (%)	185 (0.45%)	-	-	185 (2.89%)	141 (2.93%)
Lomerizine and beta-blockers, n (%)	51 (0.12%)	-	-	51 (0.80%)	43 (6.79%)
Lomerizine and anticonvulsants, n (%)	397 (0.96%)	-	-	397 (6.19%)	327 (2.66%)
Lomerizine and antidepressants, n (%)	150 (0.36%)	-	-	150 (2.34%)	128 (45.65%)
Lomerizine and verapamil, n (%)	13 (0.03%)	-	-	13 (0.20%)	13 (0.27%)
Beta-blocker and anticonvulsants, n (%)	40 (0.10%)	-	-	40 (0.39%)	31 (0.39%)
Beta-blocker and Antidepressants, n (%)	25 (0.06%)	-	-	25 (0.80%)	19 (0.04%)
Beta-blocker and verapamil, n (%)	4 (0.01%)	-	-	4 (0.06%)	2 (2.33%)
Anticonvulsants and antidepressants, n (%)	154 (0.37%)	-	-	154 (0.34%)	112 (0.06%)
Anticonvulsants and verapamil, n (%)	22 (0.05%)	-	-	22 (0.05%)	19 (0.21%)
Antidepressants and verapamil, n (%)	3 (0.01%)	-	-	3 (0.22%)	3 (0.27%)
Lomerizine, propranolol, anticonvulsants, n (%)	10 (0.02%)	-	-	10 (0.05%)	10 (2.33%)
Lomerizine, propranolol, antidepressants, n (%)	14 (0.03%)	-	-	14 (0.16%)	12 (0.39%)
Lomerizine, anticonvulsants, antidepressants, n (%)	60 (0.15%)	-	-	60 (0.94%)	55 (1.14%)
Lomerizine, anticonvulsants, verapamil, n (%)	10 (0.02%)	-	-	10 (0.16%)	10 (0.21%)
Lomerizine, antidepressants, verapamil, n (%)	1 (0.01%)	-	-	1 (0.02%)	0 (0.00%)
Propranolol, anticonvulsants, antidepressants, n (%)	25 (0.06%)	-	-	25 (0.39%)	23 (0.48%)
Proarnolol, anticonvulsants, verapamil, n (%)	2 (0.01%)	-	-	2 (0.03%)	1 (0.02%)
Anticonvulsants, antidepressants, verapamil, n (%)	2 (0.01%)	-	-	2 (0.03%)	2 (0.04%)
Lomerizine, propranolol, anticonvulsants, antidepressants, n (%)	4 (0.01%)	-	-	4 (0.06%)	4 (0.08%)
Lomerizine, propranolol, antidepressants, verapamil, n (%)	2 (0.01%)	-	-	2 (0.03%)	1 (0.02%)
Lomerizine, anticonvulsants, antidepressants, verapamil, n (%)	1 (0.01%)	-	-	1 (0.02%)	1 (0.02%)
Propranolol, anticonvulsants, antidepressants, verapamil, n (%)	2 (0.01%)	-	-	2 (0.03%)	0 (0.00%)
Lomerizine, propranolol, anticonvulsants, antidepressants, verapamil, n (%)	2 (0.01%)	-	-	2 (0.03%)	2 (0.04%)

**Table 11:** Department and treatment pattern GP: general practitioner; NSAID: nonsteroidal anti-inflammatory drug; OP: overprescription

	GP (n=4677), n (%)	Neurology (n=324), n (%)	Neurosurgery (n=607), n (%)	Gynecology (n=1773), n (%)	Orthopedics (n=866), n (%)	Psychiatry (n=795), n (%)	Others (n=1580), n (%)	Not described (n=30,587), n (%)
Single NSAID OP	1113 (23.80%)	79 (24.43%)	144 (23.77%)	418 (23.56%)	215 (24.81%)	198 (24.91%)	376 (23.80%)	6737 (22.03%)
Combination NSAIDs OP	127 (2.72%)	11 (3.26%)	17 (2.83%)	50 (2.84%)	28 (3.21%)	26 (3.21%)	37 (2.34%)	800 (2.62%)
Triptan OP	274 (5.86%)	22 (6.64%)	40 (6.60%)	98 (5.52%)	45 (5.80%)	46 (5.80%)	79 (5.00%)	1514 (4.95%)
Multiple drugs OP	446 (9.53%)	31 (9.43%)	58 (9.53%)	169 (9.52%)	81 (9.65%)	77 (9.65%)	165 (10.44%)	2990 (9.78%)
At least one of the above	1848 (39.52%)	131 (40.55%)	244 (40.25%)	696 (39.24%)	351 (41.49%)	327 (41.09%)	625 (39.56%)	11,405 (37.29%)
Prophylaxis prescription	732 (15.65%)	60 (18.55%)	101 (16.61%)	281 (15.87%)	136 (13.58%)	108 (13.58%)	224 (14.18%)	4750 (15.53%)

**Table 12:** Treatment pattern of single NSAID (expansion of Table 3) d: days; NSAIDs: nonsteroidal anti-inflammatory drugs; SD: standard deviation; tbl: tablets

Characteristics	Overall	0-11 tbl/90 d	12-29 tbl/90 d	30-44 tbl/90 d	≥45 tbl/90 d
N (%)	31251 (100.00%)	7361 (23.55%)	9262 (29.64%)	5348 (17.11%)	9280 (29.70%)
Number of tbl/90 d, mean ± SD	47.48 ± 67.2	7.94 ± 2.58	19.45 ± 4.48	36.31 ± 5.08	113.27 ± 93.5
Age, years, mean ± SD	37.4 ± 11.52	36.23 ± 11.53	36.2 ± 11.24	37.03 ± 11.08	39.73 ± 11.67
Sex: female, n (%)	22631 (72.42%)	4982 (67.68%)	6646 (71.76%)	3970 (74.23%)	7033 (75.79%)
Acute treatment, n (%)					
Single NSAIDs, n (%)	17342 (55.49%)	3783 (51.39%)	5166 (55.78%)	2963 (55.40%)	5430 (58.51%)
Combination and single NSAIDs, n (%)	1133 (3.63%)	246 (3.34%)	346 (3.74%)	193 (3.61%)	348 (3.75%)
Single NSAIDs and triptan, n (%)	11968 (38.30%)	3124 (42.44%)	3494 (37.72%)	2067 (38.65%)	3283 (35.38%)
All, n (%)	808 (2.59%)	208 (2.83%)	256 (2.76%)	125 (2.34%)	219 (2.36%)
Prophylactic treatment, n (%)	4189 (13.40%)	850 (11.55%)	1172 (12.65%)	685 (12.81%)	1482 (15.97%)
None, n (%)	27062 (86.60%)	6511 (88.45%)	8090 (87.35%)	4663 (87.19%)	7798 (84.03%)
Single, n (%)	3513 (11.24%)	753 (10.23%)	997 (10.76%)	585 (10.94%)	1178 (12.69%)
Two types, n (%)	573 (1.83%)	81 (1.10%)	148 (1.60%)	91 (1.70%)	253 (2.73%)
Three types, n (%)	93 (0.30%)	16 (0.22%)	25 (0.27%)	9 (0.17%)	43 (0.46%)
Four types, n (%)	8 (0.03%)	0 (0.00%)	2 (0.02%)	0 (0.00%)	6 (0.06%)
Five types, n (%)	2 (0.01%)	0 (0.00%)	0 (0.00%)	0 (0.00%)	2 (0.02%)
Calcium-channel blockers (lomerizine), n (%)	1754 (5.61%)	389 (5.28%)	506 (5.46%)	294 (5.50%)	565 (6.09%)
Beta-blockers (propranolol), n (%)	211 (0.68%)	35 (0.48%)	70 (0.76%)	35 (0.65%)	71 (0.77%)
Anticonvulsants (valproic acid), n (%)	1015 (3.25%)	212 (2.88%)	281 (3.03%)	152 (2.84%)	370 (3.99%)
Antidepressants (amitriptyline), n (%)	435 (1.39%)	99 (1.34%)	113 (1.22%)	80 (1.50%)	143 (1.54%)
Calcium-channel blockers (verapamil), n (%)	98 (0.31%)	18 (0.24%)	27 (0.29%)	24 (0.45%)	29 (0.31%)
Lomerizine and beta-blockers, n (%)	25 (0.08%)	3 (0.04%)	3 (0.03%)	3 (0.06%)	16 (0.17%)
Lomerizine and anticonvulsants, n (%)	267 (0.85%)	36 (0.49%)	69 (0.74%)	44 (0.82%)	118 (1.27%)
Lomerizine and antidepressants, n (%)	99 (0.32%)	13 (0.18%)	29 (0.31%)	12 (0.22%)	45 (0.48%)
Lomerizine and verapamil, n (%)	8 (0.03%)	3 (0.04%)	2 (0.02%)	0 (0.00%)	3 (0.03%)
Beta-blocker and anticonvulsants, n (%)	26 (0.08%)	3 (0.04%)	10 (0.11%)	7 (0.13%)	6 (0.06%)
Beta-blocker and antidepressants, n (%)	15 (0.05%)	4 (0.05%)	3 (0.03%)	1 (0.02%)	7 (0.08%)
Beta-blocker and verapamil, n (%)	3 (0.01%)	2 (0.03%)	1 (0.01%)	0 (0.00%)	0 (0.00%)
Anticonvulsants and antidepressants, n (%)	116 (0.37%)	14 (0.19%)	29 (0.31%)	20 (0.37%)	53 (0.57%)
Anticonvulsants and verapamil, n (%)	12 (0.04%)	3 (0.04%)	2 (0.02%)	2 (0.04%)	5 (0.05%)
Antidepressants and verapamil, n (%)	2 (0.01%)	0 (0.00%)	0 (0.00%)	2 (0.04%)	0 (0.00%)
Lomerizine, propranolol, anticonvulsants, n (%)	7 (0.02%)	0 (0.00%)	1 (0.01%)	0 (0.00%)	6 (0.06%)
Lomerizine, propranolol, antidepressants, n (%)	9 (0.03%)	1 (0.01%)	1 (0.01%)	1 (0.02%)	6 (0.06%)
Lomerizine, anticonvulsants, antidepressants, n (%)	44 (0.14%)	6 (0.08%)	15 (0.16%)	3 (0.06%)	20 (0.22%)
Lomerizine, anticonvulsants, verapamil, n (%)	10 (0.03%)	3 (0.04%)	4 (0.04%)	1 (0.02%)	2 (0.02%)
Lomerizine, antidepressants, verapamil, n (%)	1 (0.00%)	0 (0.00%)	0 (0.00%)	0 (0.00%)	1 (0.01%)
Propranolol, anticonvulsants, antidepressants, n (%)	18 (0.06%)	4 (0.05%)	2 (0.02%)	4 (0.07%)	8 (0.09%)
Proarnolol, anticonvulsants, verapamil, n (%)	2 (0.01%)	0 (0.00%)	2 (0.02%)	0 (0.00%)	0 (0.00%)
Anticonvulsants, antidepressants, verapamil, n (%)	2 (0.01%)	2 (0.03%)	0 (0.00%)	0 (0.00%)	0 (0.00%)
Lomerizine, propranolol, anticonvulsants, antidepressants, n (%)	4 (0.01%)	0 (0.00%)	0 (0.00%)	0 (0.00%)	4 (0.04%)
Lomerizine, propranolol, antidepressants, verapamil, n (%)	2 (0.01%)	0 (0.00%)	0 (0.00%)	0 (0.00%)	2 (0.02%)
Lomerizine, anticonvulsants, antidepressants, verapamil, n (%)	0 (0.00%)	0 (0.00%)	0 (0.00%)	0 (0.00%)	0 (0.00%)
Propranolol, anticonvulsants, antidepressants, verapamil, n (%)	2 (0.01%)	0 (0.00%)	2 (0.02%)	0 (0.00%)	0 (0.00%)
Lomerizine, propranolol, anticonvulsants, antidepressants, verapamil, n (%)	2 (0.01%)	0 (0.00%)	0 (0.00%)	0 (0.00%)	2 (0.02%)

**Table 13:** Treatment pattern of combination NSAIDs d: days; NSAIDs: nonsteroidal anti-inflammatory drugs; SD: standard deviation; tbl: tablets

Characteristics	Overall	0-11 tbl/90 d	12-29 tbl/90 d	30-44 tbl/90 d	≥45 tbl/90 d
N (%)	4176 (100.00%)	1866 (44.68%)	1214 (29.07%)	549 (13.15%)	547 (13.10%)
Number of tbl/90 d, mean ± SD	26.58 ± 37.82	8.15 ± 2.53	19.23 ± 4.29	35.58 ± 5.44	96.76 ± 67.44
Age, years, mean ± SD	38.14 ± 11.78	37.36 ± 11.9	38.28 ± 11.73	38.55 ± 11.73	40.04 ± 11.32
Sex: female, n (%)	2988 (71.55%)	1257 (67.36%)	907 (74.71%)	416 (75.77%)	408 (74.59%)
Acute treatment, n (%)					
Combination NSAIDs, n (%)	1029 (24.64%)	460 (24.65%)	319 (26.28%)	143 (26.05%)	107 (19.56%)
Combination and single NSAIDs, n (%)	1133 (27.13%)	501 (26.85%)	339 (27.92%)	143 (26.05%)	150 (27.42%)
Combination NSAIDs and triptan, n (%)	1206 (28.88%)	542 (29.05%)	334 (27.51%)	162 (29.51%)	168 (30.71%)
All, n (%)	808 (19.35%)	363 (19.45%)	222 (18.29%)	101 (18.40%)	122 (22.30%)
Prophylactic treatment, n (%)	929 (22.27%)	334 (17.90%)	286 (23.56%)	142 (25.87%)	168 (30.71%)
None, n (%)	3247 (77.75%)	1866 (82.10%)	928 (76.44%)	407 (74.13%)	379 (69.29%)
Single, n (%)	758 (18.15%)	305 (16.35%)	228 (18.78%)	105 (19.13%)	120 (21.94%)
Two types, n (%)	150 (3.59%)	26 (1.39%)	57 (4.70%)	33 (6.01%)	34 (6.22%)
Three types, n (%)	18 (0.43%)	3 (0.16%)	1 (0.08%)	4 (0.73%)	10 (1.83%)
Four types, n (%)	1 (0.02%)	0 (0.00%)	0 (0.00%)	0 (0.00%)	1 (0.18%)
Five types, n (%)	2 (0.05%)	0 (0.00%)	0 (0.00%)	0 (0.00%)	2 (0.37%)
Calcium-channel blockers (lomerizine), n (%)	369 (8.84%)	133 (7.13%)	130 (10.71%)	47 (8.56%)	59 (10.79%)
Beta-blockers (propranolol), n (%)	32 (0.77%)	12 (0.64%)	10 (0.82%)	5 (0.91%)	5 (0.91%)
Anticonvulsants (valproic acid), n (%)	239 (5.72%)	110 (5.89%)	55 (4.53%)	38 (6.92%)	36 (6.58%)
Antidepressants (amitriptyline), n (%)	105 (2.51%)	45 (2.41%)	31 (2.55%)	12 (2.19%)	17 (3.11%)
Calcium-channel blockers (verapamil), n (%)	13 (0.31%)	5 (0.27%)	2 (0.16%)	3 (0.55%)	3 (0.55%)
Lomerizine and beta-blockers, n (%)	6 (0.14%)	1 (0.05%)	5 (0.41%)	0 (0.00%)	0 (0.00%)
Lomerizine and anticonvulsants, n (%)	73 (1.75%)	10 (0.54%)	33 (2.72%)	15 (2.73%)	15 (2.74%)
Lomerizine and antidepressants, n (%)	26 (0.62%)	9 (0.48%)	7 (0.58%)	5 (0.91%)	5 (0.91%)
Lomerizine and verapamil, n (%)	2 (0.05%)	2 (0.11%)	0 (0.00%)	0 (0.00%)	0 (0.00%)
Beta-blocker and anticonvulsants, n (%)	10 (0.24%)	1 (0.05%)	3 (0.25%)	4 (0.73%)	2 (0.37%)
Beta-blocker and antidepressants, n (%)	3 (0.07%)	0 (0.00%)	2 (0.16%)	1 (0.18%)	0 (0.00%)
Beta-blocker and verapamil, n (%)	1 (0.02%)	1 (0.05%)	0 (0.00%)	0 (0.00%)	0 (0.00%)
Anticonvulsants and antidepressants, n (%)	26 (0.62%)	1 (0.05%)	7 (0.58%)	8 (1.46%)	10 (1.83%)
Anticonvulsants and verapamil, n (%)	3 (0.07%)	1 (0.05%)	0 (0.00%)	0 (0.00%)	2 (0.37%)
Antidepressants and verapamil, n (%)	0 (0.00%)	0 (0.00%)	0 (0.00%)	0 (0.00%)	0 (0.00%)
Lomerizine, propranolol, anticonvulsants, n (%)	3 (0.07%)	0 (0.00%)	0 (0.00%)	1 (0.18%)	2 (0.37%)
Lomerizine, propranolol, antidepressants, n (%)	1 (0.02%)	0 (0.00%)	1 (0.08%)	0 (0.00%)	0 (0.00%)
Lomerizine, anticonvulsants, antidepressants, n (%)	7 (0.17%)	2 (0.11%)	0 (0.00%)	1 (0.18%)	4 (0.73%)
Lomerizine, anticonvulsants, verapamil, n (%)	0 (0.00%)	0 (0.00%)	0 (0.00%)	0 (0.00%)	0 (0.00%)
Lomerizine, antidepressants, verapamil, n (%)	0 (0.00%)	0 (0.00%)	0 (0.00%)	0 (0.00%)	0 (0.00%)
Propranolol, anticonvulsants, antidepressants, n (%)	7 (0.17%)	1 (0.05%)	0 (0.00%)	2 (0.36%)	4 (0.73%)
Propranolol, anticonvulsants, verapamil, n (%)	0 (0.00%)	0 (0.00%)	0 (0.00%)	0 (0.00%)	0 (0.00%)
Anticonvulsants, antidepressants, verapamil, n (%)	0 (0.00%)	0 (0.00%)	0 (0.00%)	0 (0.00%)	0 (0.00%)
Lomerizine, propranolol, anticonvulsants, antidepressants, n (%)	0 (0.00%)	0 (0.00%)	0 (0.00%)	0 (0.00%)	0 (0.00%)
Lomerizine, propranolol, antidepressants, verapamil, n (%)	0 (0.00%)	0 (0.00%)	0 (0.00%)	0 (0.00%)	0 (0.00%)
Lomerizine, anticonvulsants, antidepressants, verapamil, n (%)	0 (0.00%)	0 (0.00%)	0 (0.00%)	0 (0.00%)	0 (0.00%)
Propranolol, anticonvulsants, antidepressants, verapamil, n (%)	1 (0.02%)	0 (0.00%)	0 (0.00%)	0 (0.00%)	1 (0.18%)
Lomerizine, propranolol, anticonvulsants, antidepressants, verapamil, n (%)	2 (0.05%)	0 (0.00%)	0 (0.00%)	0 (0.00%)	2 (0.37%)

**Table 14:** Treatment pattern of triptans (expansion of Table 4) d: days; NSAIDs: nonsteroidal anti-inflammatory drugs; SD: standard deviation; tbl: tablets

Characteristics	Overall	0-11 tbl/90 d	12-29 tbl/90 d	30-44 tbl/90 d	≥45 tbl/90 d
N (%)	21,704 (100.00%)	15,131 (69.72%)	4455 (20.53%)	1414 (6.51%)	704 (3.24%)
Number of tbl/90 d, mean ± SD	12.22 ± 14.12	5.82 ± 2.85	18.36 ± 4.59	34.75 ± 4.82	65.76 ± 28.04
Age, years, mean ± SD	36.58 ± 11.06	36.22 ± 11.14	37.04 ± 10.88	37.72 ± 10.62	39.18 ± 10.83
Sex: female, n (%)	15,730 (72.48%)	10,732 (70.93%)	3404 (76.41%)	1075 (76.03%)	519 (73.72%)
Acute treatment, n (%)					
Triptan, n (%)	7722 (35.58%)	5233 (34.58%)	1633 (36.66%)	552 (39.04%)	304 (43.18%)
Combination NSAIDs and triptan, n (%)	1206 (5.56%)	929 (6.14%)	205 (4.60%)	54 (3.82%)	18 (2.56%)
Single NSAIDs and triptan, n (%)	11,968 (55.14%)	8382 (55.40%)	2449 (54.97%)	752 (53.18%)	385 (54.69%)
All, n (%)	808 (3.72%)	587 (3.88%)	138 (3.10%)	56 (3.96%)	27 (3.84%)
Prophylactic treatment, n (%)	4813 (22.18%)	2409 (15.92%)	1442 (32.37%)	597 (42.22%)	365 (51.85%)
None, n (%)	16,891 (77.82%)	12,722 (84.08%)	3013 (67.63%)	817 (57.78%)	339 (48.15%)
Single, n (%)	3995 (18.41%)	2116 (13.98%)	1167 (26.20%)	456 (32.25%)	256 (36.36%)
Two types, n (%)	697 (3.21%)	265 (1.75%)	224 (5.03%)	123 (8.70%)	85 (12.07%)
Three types, n (%)	113 (0.52%)	26 (0.17%)	48 (1.08%)	17 (1.20%)	22 (3.13%)
Four types, n (%)	6 (0.03%)	2 (0.01%)	3 (0.07%)	1 (0.07%)	0 (0.00%)
Five types, n (%)	2 (0.01%)	0 (0.00%)	0 (0.00%)	0 (0.00%)	2 (0.28%)
Calcium-channel blockers (lomerizine), n (%)	2197 (10.12%)	1168 (7.72%)	643 (14.43%)	262 (18.53%)	124 (17.61%)
Beta-blockers (propranolol), n (%)	170 (0.78%)	88 (0.58%)	54 (1.21%)	13 (0.92%)	15 (2.13%)
Anticonvulsants (valproic acid), n (%)	1039 (4.79%)	538 (3.56%)	305 (6.85%)	112 (7.92%)	84 (11.93%)
Antidepressants (amitriptyline), n (%)	448 (2.06%)	249 (1.65%)	122 (2.74%)	52 (3.68%)	25 (3.55%)
Calcium-channel blockers (verapamil), n (%)	141 (0.65%)	73 (0.48%)	43 (0.97%)	17 (1.20%)	8 (1.14%)
Lomerizine and beta-blockers, n (%)	43 (0.20%)	19 (0.13%)	10 (0.22%)	6 (0.42%)	8 (1.14%)
Lomerizine and anticonvulsants, n (%)	327 (1.51%)	131 (0.87%)	96 (2.15%)	63 (4.46%)	37 (5.26%)
Lomerizine and antidepressants, n (%)	128 (0.59%)	52 (0.34%)	45 (1.01%)	17 (1.20%)	14 (1.99%)
Lomerizine and verapamil, n (%)	13 (0.06%)	6 (0.04%)	5 (0.11%)	1 (0.07%)	1 (0.14%)
Beta-blocker and anticonvulsants, n (%)	31 (0.14%)	11 (0.07%)	11 (0.25%)	4 (0.28%)	5 (0.71%)
Beta-blocker and antidepressants, n (%)	19 (0.09%)	5 (0.03%)	7 (0.16%)	5 (0.35%)	2 (0.28%)
Beta-blocker and verapamil, n (%)	2 (0.01%)	0 (0.00%)	0 (0.00%)	1 (0.07%)	1 (0.14%)
Anticonvulsants and antidepressants, n (%)	112 (0.52%)	33 (0.22%)	43 (0.97%)	21 (1.49%)	15 (2.13%)
Anticonvulsants and verapamil, n (%)	19 (0.09%)	5 (0.03%)	7 (0.16%)	5 (0.35%)	2 (0.28%)
Antidepressants and verapamil, n (%)	3 (0.01%)	3 (0.02%)	0 (0.00%)	0 (0.00%)	0 (0.00%)
Lomerizine, propranolol, anticonvulsants, n (%)	10 (0.05%)	3 (0.02%)	1 (0.02%)	5 (0.35%)	1 (0.14%)
Lomerizine, propranolol, antidepressants, n (%)	12 (0.06%)	3 (0.02%)	6 (0.13%)	0 (0.00%)	3 (0.43%)
Lomerizine, anticonvulsants, antidepressants, n (%)	55 (0.25%)	12 (0.08%)	25 (0.56%)	4 (0.28%)	14 (1.99%)
Lomerizine, anticonvulsants, verapamil, n (%)	10 (0.05%)	2 (0.01%)	6 (0.13%)	1 (0.07%)	1 (0.14%)
Lomerizine, antidepressants, verapamil, n (%)	0 (0.00%)	0 (0.00%)	0 (0.00%)	0 (0.00%)	0 (0.00%)
Propranolol, anticonvulsants, antidepressants, n (%)	23 (0.11%)	5 (0.03%)	10 (0.22%)	7 (0.50%)	1 (0.14%)
Proarnolol, anticonvulsants, verapamil, n (%)	1 (0.00%)	1 (0.01%)	0 (0.00%)	0 (0.00%)	0 (0.00%)
Anticonvulsants, antidepressants, verapamil, n (%)	2 (0.01%)	0 (0.00%)	0 (0.00%)	0 (0.00%)	2 (0.28%)
Lomerizine, propranolol, anticonvulsants, antidepressants, n (%)	4 (0.02%)	0 (0.00%)	3 (0.07%)	1 (0.07%)	0 (0.00%)
Lomerizine, propranolol, antidepressants, verapamil, n (%)	1 (0.00%)	1 (0.01%)	0 (0.00%)	0 (0.00%)	0 (0.00%)
Lomerizine, anticonvulsants, antidepressants, verapamil, n (%)	1 (0.00%)	1 (0.01%)	0 (0.00%)	0 (0.00%)	0 (0.00%)
Propranolol, anticonvulsants, antidepressants, verapamil, n (%)	0 (0.00%)	0 (0.00%)	0 (0.00%)	0 (0.00%)	0 (0.00%)
Lomerizine, propranolol, anticonvulsants, antidepressants, verapamil, n (%)	2 (0.01%)	0 (0.00%)	0 (0.00%)	0 (0.00%)	2 (0.28%)

**Table 15:** Treatment pattern of overprescribed any combination of single, combination, and/or triptans as well as that does not constitute overprescription for a single drug d: days; NSAIDs: nonsteroidal anti-inflammatory drugs; SD: standard deviation; tbl: tablets

Characteristics	Overall	30-44 tbl/90 d	45-59 tbl/90 d	60-72 tbl/90 d
N (%)	4016 (100.00%)	2652 (66.04%)	1193 (29.71%)	171 (4.26%)
Number of tbl/90 d, mean ± SD	41.16 ± 8.84	35.8 ± 4.38	49.79 ± 3.8	62.91 ± 3.06
Age, years, mean ± SD	36.72 ± 10.87	36.78 ± 10.86	36.46 ± 10.85	37.78 ± 11.26
Sex: female, n (%)	2985 (74.33%)	1944 (7330.00%)	920 (77.12%)	121 (70.76%)
Acute treatment, n (%)				
Combination and single NSAIDs, n (%)	341 (8.49%)	216 (8.14%)	105 (8.80%)	20 (11.70%)
Single NSAIDs and triptan, n (%)	3244 (80.78%)	2144 (80.84%)	966 (80.97%)	134 (78.36%)
Combination NSAIDs and triptan, n (%)	143 (3.56%)	129 (4.86%)	14 (1.17%)	0 (0.00%)
All, n (%)	288 (7.17%)	163 (6.15%)	108 (9.05%)	17 (9.94%)
Prophylactic treatment, n (%)	860 (21.41%)	533 (20.10%)	281 (23.55%)	46 (26.90%)
None, n (%)	3156 (78.59%)	2119 (79.90%)	912 (76.45%)	125 (73.10%)
Single, n (%)	723 (18.00%)	452 (17.04%)	229 (19.20%)	42 (24.56%)
Two types, n (%)	117 (2.91%)	69 (2.60%)	45 (3.77%)	3 (1.75%)
Three types, n (%)	20 (0.50%)	12 (0.45%)	7 (0.59%)	1 (0.58%)
Four types, n (%)	0 (0.00%)	0 (0.00%)	0 (0.00%)	0 (0.00%)
Five types, n (%)	0 (0.00%)	0 (0.00%)	0 (0.00%)	0 (0.00%)
Calcium-channel blockers (lomerizine), n (%)	408 (10.16%)	255 (9.62%)	128 (10.73%)	25 (14.62%)
Beta-blockers (propranolol), n (%)	40 (1.00%)	23 (0.87%)	16 (1.34%)	1 (0.58%)
Anticonvulsants (valproic acid), n (%)	177 (4.41%)	111 (4.19%)	55 (4.61%)	11 (6.43%)
Antidepressants (amitriptyline), n (%)	79 (1.97%)	50 (1.89%)	26 (2.18%)	3 (1.75%)
Calcium-channel blockers (verapamil), n (%)	19 (0.47%)	13 (0.49%)	4 (0.34%)	2 (1.17%)
Lomerizine and beta-blockers, n (%)	2 (0.05%)	0 (0.00%)	2 (0.17%)	0 (0.00%)
Lomerizine and anticonvulsants, n (%)	50 (1.25%)	34 (1.28%)	14 (1.17%)	2 (1.17%)
Lomerizine and antidepressants, n (%)	23 (0.57%)	10 (0.38%)	13 (1.09%)	0 (0.00%)
Lomerizine and verapamil, n (%)	2 (0.05%)	2 (0.08%)	0 (0.00%)	0 (0.00%)
Beta-blocker and anticonvulsants, n (%)	8 (0.20%)	5 (0.19%)	2 (0.17%)	1 (0.58%)
Beta-blocker and antidepressants, n (%)	4 (0.10%)	2 (0.08%)	2 (0.17%)	0 (0.00%)
Beta-blocker and verapamil, n (%)	0 (0.00%)	0 (0.00%)	0 (0.00%)	0 (0.00%)
Anticonvulsants and antidepressants, n (%)	24 (0.60%)	14 (0.53%)	10 (0.84%)	0 (0.00%)
Anticonvulsants and verapamil, n (%)	2 (0.05%)	2 (0.08%)	0 (0.00%)	0 (0.00%)
Antidepressants and verapamil, n (%)	2 (0.05%)	0 (0.00%)	2 (0.17%)	0 (0.00%)
Lomerizine, propranolol, anticonvulsants, n (%)	0 (0.00%)	0 (0.00%)	0 (0.00%)	0 (0.00%)
Lomerizine, propranolol, antidepressants, n (%)	1 (0.02%)	0 (0.00%)	1 (0.08%)	0 (0.00%)
Lomerizine, anticonvulsants, antidepressants, n (%)	12 (0.30%)	6 (0.23%)	5 (0.42%)	1 (0.58%)
Lomerizine, anticonvulsants, verapamil, n (%)	5 (0.12%)	4 (0.15%)	1 (0.08%)	0 (0.00%)
Lomerizine, antidepressants, verapamil, n (%)	0 (0.00%)	0 (0.00%)	0 (0.00%)	0 (0.00%)
Propranolol, anticonvulsants, antidepressants, n (%)	2 (0.05%)	2 (0.08%)	0 (0.00%)	0 (0.00%)
Propranolol, anticonvulsants, verapamil, n (%)	0 (0.00%)	0 (0.00%)	0 (0.00%)	0 (0.00%)
Anticonvulsants, antidepressants, verapamil, n (%)	0 (0.00%)	0 (0.00%)	0 (0.00%)	0 (0.00%)
Lomerizine, propranolol, anticonvulsants, antidepressants, n (%)	0 (0.00%)	0 (0.00%)	0 (0.00%)	0 (0.00%)
Lomerizine, propranolol, antidepressants, verapamil, n (%)	0 (0.00%)	0 (0.00%)	0 (0.00%)	0 (0.00%)
Lomerizine, anticonvulsants, antidepressants, verapamil, n (%)	0 (0.00%)	0 (0.00%)	0 (0.00%)	0 (0.00%)
Propranolol, anticonvulsants, antidepressants, verapamil, n (%)	0 (0.00%)	0 (0.00%)	0 (0.00%)	0 (0.00%)
Lomerizine, propranolol, anticonvulsants, antidepressants, verapamil, n (%)	0 (0.00%)	0 (0.00%)	0 (0.00%)	0 (0.00%)

**Table 16:** Cox regression analyses results for 90-day overprescription (one-term) (n=217,246) (expansion of Table 8) ADI: area deprivation index; B: slope; d: days; NSAIDs: nonsteroidal anti-inflammatory drugs; SD: standard deviation; tbl: tablets

Overprescription of any type of medication	B	Standard error	Wald	P-value	Odds ratio	95% CI lower	95% CI upper
Female	0.16	0.01	450.55	<0.001	1.17	1.15	1.18
Age (y.o.)	0.01	0.00	2034.56	<0.001	1.01	1.01	1.01
Single NSAID during the first 90 d (tbl/90 d)	0.00	0.00	207.60	<0.001	1.00	1.00	1.00
Combination NSAIDs during the first 90 d (tbl/90 d)	0.00	0.00	134.03	<0.001	1.00	1.00	1.00
Triptan during the first 90 d (tbl/90 d)	0.00	0.00	1130.40	<0.001	1.00	1.00	1.00
Presence of prophylactic treatment prescription during the first 90 d	0.26	0.01	953.97	<0.001	1.30	1.28	1.32
ADI	0.01	0.01	1.21	0.271	1.01	0.99	1.02
The number of headache specialists (/100,000 people)	-0.04	0.01	9.50	0.002	0.97	0.94	0.99
Single NSAID	B	Standard error	Wald	P-value	Odds ratio	95% CI lower	95% CI upper
Female	0.17	0.01	332.38	<0.001	1.19	1.17	1.21
Age (y.o.)	0.019	0.00	3131.61	<0.001	1.02	1.02	1.02
Single NSAID during the first 90 d (tbl/90 d)	0.00	0.00	376.71	<0.001	1.00	1.00	1.00
Combination NSAIDs during the first 90 d (tbl/90 d)	0.00	0.00	16.91	<0.001	1.00	1.00	1.00
Triptan during the first 90 d (tbl/90 d)	0.00	0.00	2.52	0.113	1.00	1.00	1.00
Presence of prophylactic treatment prescription during the first 90 d	0.152	0.11	178.15	<0.001	1.16	1.14	1.12
ADI	0.084	0.01	76.20	<0.001	1.09	1.07	1.11
The number of headache specialists (/100,000 people)	-0.06	0.02	13.32	<0.001	0.95	0.92	0.98
Combination NSAIDs	B	Standard error	Wald	P-value	Odds ratio	95% CI lower	95% CI upper
Female	0.22	0.03	48.48	<0.001	1.25	1.17	1.33
Age (y.o.)	0.02	0.00	338.41	<0.001	1.02	1.02	1.02
Single NSAID during the first 90 d (tbl/90 d)	0.00	0.00	0.00	0.962	1.00	1.00	1.00
Combination NSAIDs during the first 90 d (tbl/90 d)	0.00	0.00	709.28	<0.001	1.00	1.00	1.00
Triptan during the first 90 d (tbl/90 d)	0.00	0.00	0.24	0.625	1.00	1.00	1.00
Presence of prophylactic treatment prescription during the first 90 d	0.51	0.03	224.57	<0.001	1.66	1.56	1.78
ADI	-0.32	0.03	86.83	<0.001	0.73	0.68	0.78
The number of headache specialists (/100,000 people)	-0.54	0.05	100.55	<0.001	0.58	0.53	0.65
Triptan	B	Standard error	Wald	P-value	Odds ratio	95% CI lower	95% CI upper
Female	0.11	0.02	22.01	<0.001	1.12	1.07	1.17
Age (y.o.)	0.01	0.00	90.77	<0.001	1.01	1.01	1.01
Single NSAID during the first 90 d (tbl/90 d)	0.00	0.00	1.58	0.21	1.00	1.00	1.00
Combination NSAIDs during the first 90 d (tbl/90 d)	0.00	0.00	3.36	0.07	1.00	1.00	1.00
Triptan during the first 90 d (tbl/90 d)	0.00	0.00	3383.14	<0.001	1.00	1.00	1.00
Presence of prophylactic treatment prescription during the first 90 d	1.34	0.02	3996.90	<0.001	3.81	3.66	3.97
ADI	-0.12	0.03	23.68	<0.001	0.88	0.84	0.93
The number of headache specialists (/100,000 people)	0.01	0.04	0.04	0.84	1.01	0.94	1.08
Overprescribed any combination of single, combination, and/or triptans as well as that do not constitute overprescription for a single drug	B	Standard error	Wald	P-value	Odds ratio	95% CI lower	95% CI upper
Female	0.20	0.01	379.91	<0.001	1.22	1.19	1.24
Age (y.o.)	0.00	0.00	131.16	<0.001	1.00	1.00	1.01
Single NSAID during the first 90 d (tbl/90 d)	0.00	0.00	3.34	0.067	1.00	1.00	1.00
Combination NSAIDs during the first 90 d (tbl/90 d)	0.00	0.00	0.00	0.976	1.00	1.00	1.00
Triptan during the first 90 d (tbl/90 d)	0.00	0.00	149.21	<0.001	1.00	1.00	1.00
Presence of prophylactic treatment prescription during the first 90 d	0.14	0.01	126.68	<0.001	1.15	1.12	1.17
ADI	-0.05	0.01	20.34	<0.001	0.95	0.93	0.97
The number of headache specialists (/100,000 people)	0.03	0.02	4.29	0.038	1.03	1.00	1.07
Prophylactic medication	B	Standard error	Wald	P-value	Odds ratio	95% CI lower	95% CI upper
Female	-0.06	0.01	30.84	<0.001	0.94	0.92	0.96
Age (y.o.)	0.00	0.00	110.86	<0.001	1.00	0.99	1.00
Single NSAID during the first 90 d (tbl/90 d)	0.00	0.00	0.04	0.837	1.00	1.00	1.00
Combination NSAIDs during the first 90 d (tbl/90 d)	0.00	0.00	9.51	0.002	1.00	1.00	1.00
Triptan during the first 90 d (tbl/90 d)	0.00	0.00	1659.95	<0.001	1.00	1.00	1.00
ADI	-0.05	0.01	16.49	<0.001	0.95	0.93	0.97
The number of headache specialists (/100,000 people)	0.23	0.02	149.13	<0.001	1.26	1.21	1.30

**Table 17:** Cox regression analyses results for 180-day overprescription (two-term) (n=217,246) ADI: area deprivation index; B: slope; d: days; NSAIDs: nonsteroidal anti-inflammatory drugs; SD: standard deviation; tbl: tablets

Overprescription of any type of medication	B	Standard error	Wald	P-value	Odds ratio	95% CI lower	95% CI upper
Female	0.28	0.01	411.23	<0.001	1.32	1.28	1.35
Age (y.o.)	0.03	0.00	3589.57	<0.001	1.03	1.03	1.03
Single NSAID during the first 90 d (tbl/90 d)	0.00	0.00	123.93	<0.001	1.00	1.00	1.00
Combination NSAIDs during the first 90 d (tbl/90 d)	0.00	0.00	127.03	<0.001	1.00	1.00	1.00
Triptan during the first 90 d (tbl/90 d)	0.00	0.00	718.46	<0.001	1.00	1.00	1.00
Presence of prophylactic treatment prescription during the first 90 d	0.49	0.01	1132.41	<0.001	1.62	1.58	1.67
ADI	0.01	0.01	0.16	0.686	1.01	0.98	1.03
The number of headache specialists (/100,000 people)	-0.07	0.02	12.52	<0.001	0.93	0.89	0.97
Single NSAID	B	Standard error	Wald	P-value	Odds ratio	95% CI lower	95% CI upper
Female	0.21	0.02	138.40	<0.001	1.24	1.19	1.28
Age (y.o.)	0.04	0.00	3777.07	<0.001	1.04	1.04	1.04
Single NSAID during the first 90 d (tbl/90 d)	0.00	0.00	184.05	<0.001	1.00	1.00	1.00
Combination NSAIDs during the first 90 d (tbl/90 d)	0.00	0.00	18.14	<0.001	1.00	1.00	1.00
Triptan during the first 90 d (tbl/90 d)	0.00	0.00	64.94	<0.001	1.00	1.00	1.00
Presence of prophylactic treatment prescription during the first 90 d	0.39	0.02	399.11	<0.001	1.48	1.43	1.54
ADI	0.10	0.02	30.21	<0.001	1.10	1.07	1.14
The number of headache specialists (/100,000 people)	-0.12	0.03	16.86	<0.001	0.89	0.84	0.94
Combination NSAIDs	B	Standard error	Wald	P-value	Odds ratio	95% CI lower	95% CI upper
Female	0.30	0.06	25.54	<0.001	1.34	1.20	1.51
Age (y.o.)	0.03	0.00	257.40	<0.001	1.03	1.03	1.04
Single NSAID during the first 90 d (tbl/90 d)	0.00	0.00	0.06	<0.001	1.00	1.00	1.00
Combination NSAIDs during the first 90 d (tbl/90 d)	0.00	0.00	359.09	<0.001	1.00	1.00	1.00
Triptan during the first 90 d (tbl/90 d)	0.00	0.00	2.77	<0.001	1.00	1.00	1.00
Presence of prophylactic treatment prescription during the first 90 d	0.71	0.06	152.42	<0.001	2.03	1.82	2.27
ADI	-0.24	0.06	15.84	<0.001	0.79	0.70	0.89
The number of headache specialists (/100,000 people)	-0.45	0.09	22.39	<0.001	0.64	0.53	0.77
Triptan	B	Standard error	Wald	P-value	Odds ratio	95% CI lower	95% CI lower upper
Female	0.36	0.05	60.09	<0.001	1.43	1.30	1.56
Age (y.o.)	0.02	0.00	91.29	<0.001	1.02	1.01	1.02
Single NSAID during the first 90 d (tbl/90 d)	0.00	0.00	4.38	0.036	1.00	1.00	1.00
Combination NSAIDs during the first 90 d (tbl/90 d)	0.00	0.00	2.43	0.119	1.00	1.00	1.00
Triptan during the first 90 d (tbl/90 d)	0.00	0.00	1069.81	<0.001	1.00	1.00	1.00
Presence of prophylactic treatment prescription during the first 90 d	1.26	0.04	1012.99	<0.001	3.53	3.27	3.82
ADI	-0.13	0.05	7.31	0.007	0.88	0.81	0.97
The number of headache specialists (/100,000 people)	-0.12	0.07	2.91	0.088	0.89	0.77	1.02
Overprescribed any combination of single, combination, and/or triptans as well as that do not constitute overprescription for a single drug	B	Standard error	Wald	P-value	Odds ratio	95% CI lower	95% CI upper
Female	0.46	0.04	163.07	<0.001	1.584	1.476	1.699
Age (y.o.)	0.01	0.00	131.09	<0.001	1.014	1.012	1.016
Single NSAID during the first 90 d (tbl/90 d)	0.00	0.00	0.28	0.597	1.000	1.000	1.000
Combination NSAIDs during the first 90 d (tbl/90 d)	0.00	0.00	0.02	0.898	1.000	1.000	1.000
Triptan during the first 90 d (tbl/90 d)	0.00	0.00	62.38	<0.001	1.001	1.001	1.002
Presence of prophylactic treatment prescription during the first 90 d	0.31	0.04	68.57	<0.001	1.365	1.268	1.470
ADI	-0.08	0.03	5.07	0.024	0.924	0.863	0.990
The number of headache specialists (/100,000 people)	0.08	0.05	2.45	0.117	1.085	0.980	1.203
Prophylactic medication	B	Standard error	Wald	P-value	Odds ratio	95% CI lower	95% CI upper
Female	0.17	0.02	83.42	<0.001	1.19	1.14	1.23
Age (y.o.)	0.00	0.00	0.05	0.819	1.00	1.00	1.00
Single NSAID during the first 90 d (tbl/90 d)	0.00	0.00	0.96	0.328	1.00	1.00	1.00
Combination NSAIDs during the first 90 d (tbl/90 d)	0.00	0.00	10.10	0.001	1.00	1.00	1.00
Triptan during the first 90 d (tbl/90 d)	0.00	0.00	1379.30	<0.001	1.00	1.00	1.00
ADI	-0.08	0.02	15.84	<0.001	0.93	0.89	0.96
The number of headache specialists (/100,000 people)	0.18	0.03	39.32	<0.001	1.20	1.13	1.27
